# Effects of improved drinking water quality on early childhood growth in rural Uttar Pradesh, India: A propensity-score analysis

**DOI:** 10.1371/journal.pone.0209054

**Published:** 2019-01-08

**Authors:** Mira Johri, Marie-Pierre Sylvestre, Georges Karna Koné, Dinesh Chandra, S. V. Subramanian

**Affiliations:** 1 Centre de Recherche du Centre Hospitalier de l’Université de Montréal (CRCHUM), Montreal, Québec, Canada; 2 Département d’administration de la santé, École de santé publique, Université de Montréal, Montreal, Canada; 3 Département de médicine sociale et préventive, École de santé publique, Université de Montréal, Montreal, Canada; 4 Abt associates, Health finance and governance (FHG) Project, Port-au-Prince, Haiti; 5 Independent consultant, New Delhi, India; 6 Harvard Center for Population and Development Studies, Cambridge MA, United States of America; Kansas State University, UNITED STATES

## Abstract

**Context:**

Recent randomised controlled trials in Bangladesh and Kenya concluded that household water treatment, alone or in combination with upgraded sanitation and handwashing, did not reduce linear growth faltering or improve other child growth outcomes. Whether these results are applicable in areas with distinct constellations of water, sanitation and hygiene (WaSH) risks is unknown. Analysis of observational data offers an efficient means to assess the external validity of trial findings. We studied whether a water quality intervention could improve child growth in a rural Indian setting with higher levels of circulating pathogens than the original trial sites.

**Methods:**

We analysed a cross-sectional dataset including a microbiological measure of household water quality. All households accessed water from an improved source. We applied propensity score methods to emulate a randomised trial investigating the hypothesis that receipt of drinking water meeting Sustainable Development Goal (SDG) 6.1 quality standards for absence of faecal contamination leads to improved growth. Growth outcomes (stunting, underweight, wasting, and their corresponding Z-scores) were assessed in children 12–23 months of age. For each outcome, we estimated the mean and 95% confidence interval of the absolute risk difference between treatment groups.

**Findings:**

Of 1088 households, 442 (40.62%) received drinking water meeting SDG 6.1 standards. The adjusted risk of child underweight was 7.4% (1.3% to 13.4%) lower among those drinking water satisfying SDG 6.1 norms than among controls. Evidence concerning the relationship of drinking water meeting SDG 6.1 norms to length-for-age and weight-for-age was inconclusive, and there was no apparent relationship with stunting or wasting.

**Conclusions:**

In contexts characterised by high pathogen transmission, water quality improvements have the potential to reduce the proportion of underweight children, but are unlikely to impact stunting or wasting. Further research is required to assess how these modelled benefits can best be achieved in real world settings.

## Introduction

Ending malnutrition is a critical prerequisite for sustainable development. Despite important global progress since the year 2000, undernutrition in early life remains implicated in nearly half of deaths among children under 5 globally, representing a loss of nearly 3 million child lives per year.[1, 2] Stunting (suboptimal linear growth, defined as Z-scores falling below 2 standard deviations (SD) from the length-for-age/height-for-age WHO Child Growth Standards median) is the most prevalent form of child malnutrition, affecting an estimated 150.8 million children under age 5 (22.2 percent) worldwide in 2017.[2, 3] Child undernutrition, especially stunting, is linked to poor health and developmental trajectories in early life, and lower educational attainment, shorter stature, higher risk of non-communicable diseases, and reduced earnings in adulthood.[1, 4, 5]

In recent years, the attention of researchers and international development partners has been drawn to the role of environmental contamination as a possible structural barrier to healthy development for children living in conditions of poverty. It has been hypothesised that exposure to faecal contamination in children’s living environments due to living in poor water, sanitation, and hygiene (WaSH) conditions may play a fundamental role in the genesis and persistence of childhood undernutrition.[6, 7] Theory and biological evidence suggest that a subclinical condition known as “environmental enteropathy”—alterations in the gut and immune system due to repeat exposures to pathogens related to poor WaSH—may be particularly important for chronic outcomes such linear growth faltering.[6, 8, 9] Epidemiological evidence shows that the burden of childhood growth faltering is heavily concentrated in areas of deep poverty and poor WaSH; stunting prevalences are highest in South Asia, Eastern and Southern Africa, and Western and Central Africa.[2] The UN Sustainable Development Goals (SDGs) include ambitious new targets to eliminate open defecation and achieve universal access to safely managed sanitation and drinking water services by 2030.[10]

New experimental evidence raises fundamental challenges to this paradigm. The WASH Benefits trials were parallel cluster-randomised trials conducted in Bangladesh[11] and Kenya[12] to study whether simple interventions to improve water quality, sanitation, and hand hygiene, alone or in combination with nutrition interventions, reduced diarrhoea or growth faltering. Employing a factorial design, the trials assigned pregnant women in geographically adjacent clusters by block randomisation to one of seven study groups: chlorinated drinking water (water); upgraded sanitation (sanitation); promotion of handwashing with soap (handwashing); combined water, sanitation, and handwashing; counselling on appropriate child nutrition plus supplements (nutrition); combined water, sanitation, handwashing, and nutrition; and control. Findings based on outcomes measured at 1 and 2 years of follow up revealed that water, sanitation, and handwashing interventions, singly or in combination, did not improve linear growth faltering or other growth outcomes among children born to enrolled pregnant women.[11, 12] Nutritional counselling and supplementation had modest benefits.[11, 12]

The WASH Benefits trials were well designed and conducted, with high intervention adherence particularly in Bangladesh.[11] The internal validity of the findings is likely to be high, reflecting a true lack of intervention effect in these settings.[11] However, context may be important for WaSH interventions and questions have been raised concerning the applicability of these findings in areas with distinct constellations of WaSH risks.[11, 13, 14] The WASH Benefits sites in Kenya and Bangladesh had very low prevalence of open defecation (approximately 5%), widespread access to sanitation facilities, widespread access to an improved drinking water source and, in Bangladesh, unexpectedly low diarrhoea prevalence (6% in the Bangladeshi control group).[11, 12, 14] The WASH Benefits findings are particularly salient because the interventions studied in the WASH Benefits trial–simple latrines, point-of-use water treatment, handwashing facilities—are cornerstones of WaSH intervention strategies in developing countries and of the UN SDG strategies for drinking water (Target 6.1) and sanitation and hygiene (Target 6.2).[15] It is possible that similar WaSH interventions would have a beneficial effect in populations where water access is problematic[14] or background levels of pathogen transmission are higher.[11–14, 16]

Randomised trial data are not available for all settings and judicious use of observational data can offer an efficient means to investigate the external validity of trial findings. We aimed to study whether water quality improvements similar to those studied in the WASH Benefits trial could improve early childhood growth in a rural Indian population grappling with poor living conditions, high population density, and very high prevalences of open defecation, infectious diseases and malnutrition.[17–19] Water quality intervention strategies are particularly salient for this population. While community sanitation may hold the greatest promise to reduce harmful contamination,[20–23] improvements in community infrastructure and sanitation practices are beyond the control of individuals and households and have proven difficult to modify in rural India.[24–26] Point-of-use options for water treatment such as boiling, chlorination, solar disinfection, flocculation, and filtration are low-cost, feasible strategies that could empower households to reduce exposure to contaminants in advance of major infrastructure changes.[27]

Our analysis leverages an observational dataset containing a measure of microbial drinking water quality. The effect of a simple point-of-use intervention for household treatment of drinking water similar to that used in the WASH Benefits trial would be to improve microbial water quality. We rebalanced data on observed differences in water quality using propensity scores to emulate a randomised trial investigating the hypothesis that receipt of improved drinking water free of faecal contamination leads to improved child growth in this setting.

## Methods

### Study design, participants, and sampling

Field data collection methods have been previously described.[18, 28] Briefly, from May 14th to July 13th, 2013, we conducted a cross-sectional survey in a rural district (Hardoi) of Uttar Pradesh state with poor health indicators. [S1 Table] All women who lived in rural Hardoi district and were mothers of a child aged 12 to 23 months were eligible to participate. We excluded one woman unable to communicate in Hindi or Urdu.

The sampling unit was the household. We sampled one mother per household, and one child per mother, the youngest child in the age group 12–23 months. We used two-stage probability-proportional-to-size cluster sampling to identify eligible villages, and random sampling within villages to identify eligible households. Survey size was calculated relative to the main hypothesis concerning maternal health literacy and child vaccination.[28]

The Pratham Ethics Committee (New Delhi, India) and the Research Ethics Committee of the CHUM (Comité d’éthique de la recherche du CHUM, Montréal, Canada, #12.391) approved this study. All participants gave written informed consent before participating.

### Study context: WaSH service levels

UN WaSH ladders are normative standards developed by the WHO/UNICEF Joint Monitoring Program (JMP) to portray WaSH access levels and guide service delivery improvements. For the SDG period, household service levels are categorized using five-rung ladders for water (surface water, unimproved, limited, basic, safely managed) and sanitation (open defecation, unimproved, limited, basic, and safely managed).[10] As attempts to relate learnings on the effects of WaSH interventions can be expressed in terms of changes in service levels reflecting WaSH ladder rungs,[29, 30] we characterised our analysis in relation to these service levels.

The Millennium Development Goals (MDG) period water ladder defined the highest household service level as water accessed via provenance from an “improved source”, a diverse category that includes channels such as piped water on premises, public taps and hand pumps, boreholes or tube wells, protected dug wells, protected springs, and rainwater.[10] Improved water sources are on average less contaminated than others[31, 32]; however, provenance from an improved source does not ensure water safety.[31] The highest SDG water ladder rung reflects the more ambitious “safely managed” drinking water service, which requires that households must use an “improved” drinking water source (the MDG indicator) that is additionally “located on premises, available when needed, and free of faecal and priority chemical contamination.”[33] If any one of these criteria is not met but a round trip to collect water takes 30 minutes or less, the household access level is to be classified as a “basic” drinking water service; else, it will be classified as a “limited” water service. To implement the new quality standard, the SDG 6.1 indicator monitors absence of faecal contamination in drinking water (no *E*. *coli* in 100 mL) based on microbiological testing.[33]

In our study setting, water is from an improved source and conveniently accessible to households; the minimum service level in our sample thus approximates the “basic” water rung.[17] Those households that in addition receive water free from faecal contamination achieve a service level that approximates the highest “safely managed” drinking water rung. Open defecation predominates, representing the lowest rung on the sanitation ladder.[17]

### Variables

The analysis focused on three indicators of child nutritional status: stunting, underweight, and wasting in children aged 12–23 months. We selected these outcomes as they could be linked to recurrent exposure to faecal pathogens via poor WaSH conditions typical of rural households living in poverty.[6] The exposure variable was household drinking water meeting SDG 6 standards for “safely managed” water.[34] We viewed water quality as potentially linked to nutritional outcomes through clinical (e.g. diarrhoea, other infectious diseases) and subclinical (environmental enteropathy) pathways.[8, 9, 35] We considered the following variables as potential confounders: community proportions of open defecation[20] and poverty, household assets, household access to improved sanitation[22], religion of household head, and maternal and paternal education levels. Additional variables (child age, child sex, child birth order) were viewed as possible predictors of the outcomes. These relationships are based on the scientific literature and represented in a causal diagram (S2 Fig and S1 Methods).

### Data sources and measurement

#### Outcomes

Data on children’s age, height and weight were collected by standard procedures[18, 36] and used to calculate Z-scores of length-for-age, weight-for-age, and weight-for-length.[37] We classified children as stunted, underweight, or wasted if their Z-score was less than minus two standard deviations (-2SD) from the WHO Multicentre Growth Reference Study population for children of their age and gender.[36]

#### Exposure variable

The exposure variable was defined in two stages based on the UN JMP water ladder for the SDG period.[10] First, participant responses were used to define a binary variable that distinguished households drinking water from improved versus unimproved sources. Second, we collected a drinking water sample from each household by asking: “Could you please provide me with a glass of the water that members of your household usually drink?” and tested it using a UNICEF-validated rapid test for presence of faecal indicator bacteria.[38] Households that received drinking water from an improved source free of faecal contamination were defined as receiving “safely managed” water satisfying SDG 6.1 norms.[33]

#### Potential confounders and additional predictors

Variables relating to households, mothers, and children were assessed by respondent report, except for dwelling characteristics assessed by observation. Child age was recorded from the child’s health card if available.

We recorded the highest number of years of education completed by the child’s mother and father. As respondents often reported number of years of education approximately, maternal and paternal years of education were coded into four categories to enhance reliability. We performed principal components analysis to construct a relative index of household wealth from a list of assets developed from India’s major national surveys and used this index to divide the sample into quintiles. We used restricted cubic splines to model the continuous variable child age (days). Sanitation definitions followed UN JMP norms. Prior to 2015 when our survey was fielded, the UN JMP measured access to sanitation by the percentage of the population using “improved” sanitation facilities that hygienically separate human excreta from human contact.[39] Empirically, household sanitation levels in our sample reflected either improved sanitation (highest rung on the 4-step JMP ladder) or open defecation (lowest rung on the ladder). Therefore, for this analysis, household sanitation is a binary variable representing improved sanitation versus open defecation. Because presence of physical sanitation infrastructure does not guarantee use, we also asked about sanitation practices. Proportions of community open defecation and poverty were constructed by aggregating household responses to questions concerning household sanitation practices and household assets, respectively.

### Measures to address potential biases

To minimise information and measurement bias, data collection personnel received rigorous training and were not informed about study hypotheses relating to water quality and child health. The water quality test was selected due to its ease of use and excellent performance under field conditions, yielding over 90% true results when read at 48 hours.[40] Test interpretation was performed at 48 hours by survey teams and verified by field supervisors. Duplicate measurements were taken in the case of any ambiguous test reading; values were recorded only from tests with clear results.

### Statistical methods

We visualised this observational study as a hypothetical experiment in which individuals were randomly assigned to receive either “treatment” or “control” interventions. To permit a focussed comparison, we restricted the sample to households drinking water from an improved source.[10] The “treatment” group received drinking water from an improved source free of faecal contamination (a proxy for the result of successful water treatment, approximating a “safely managed” service level meeting SDG 6.1 norms), while “controls” received drinking water from an improved source that failed to meet SDG 6.1 water quality norms due to presence of faecal contamination (approximating a “basic” water service level). We used propensity score techniques to rebalance the sample to ensure similarity between treated and control subjects in the distribution of relevant observed covariates, thereby permitting unbiased assessment of the treatment effect on child growth outcomes (stunting, underweight, wasting), under the hypothesis of no unobserved confounding.

**Fig 1:**
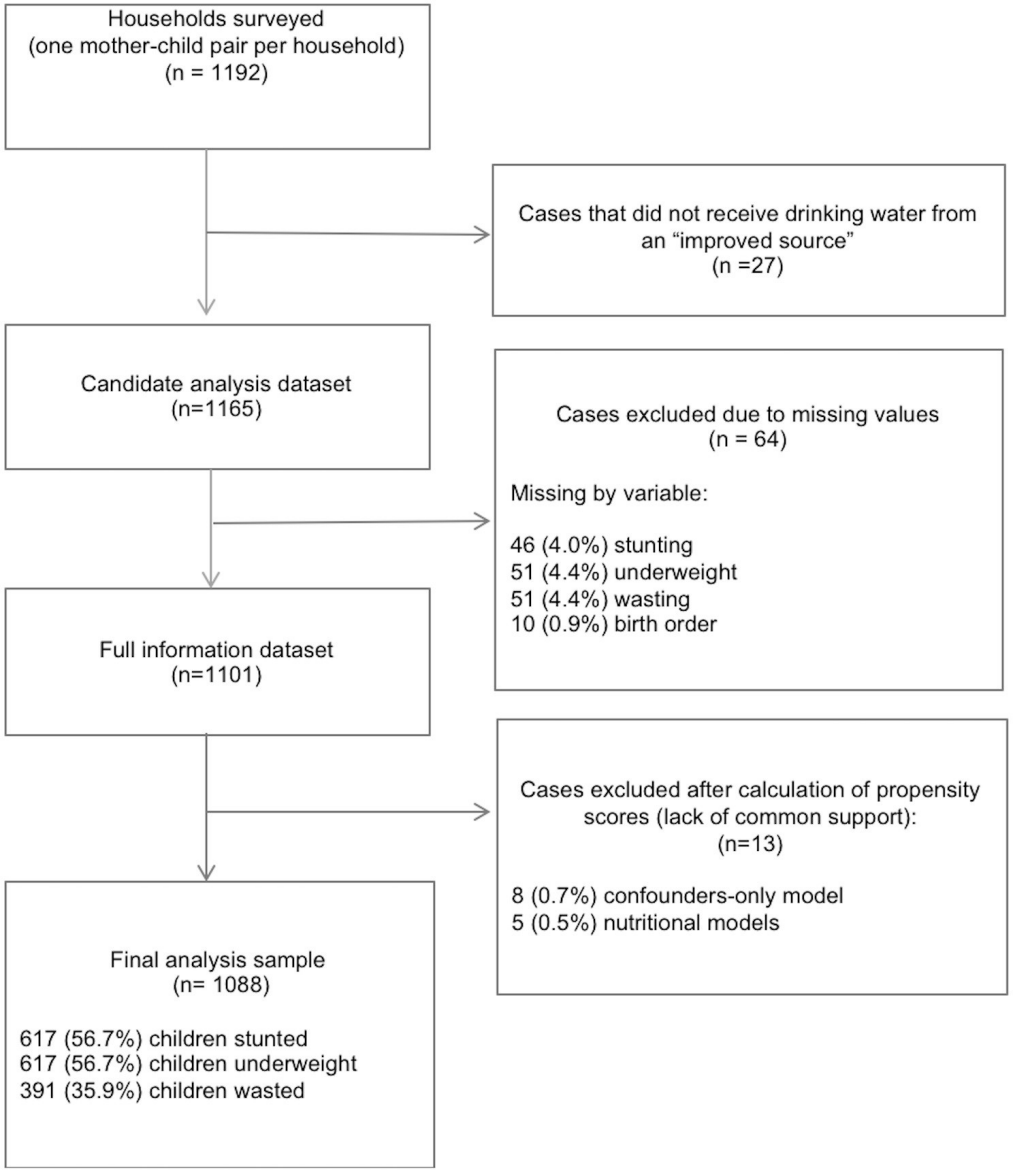
Flow diagram showing process for inclusion in data analysis.

The propensity score was estimated by logistic regression. The dependent variable was a binary indicator signalling whether household drinking water quality met SDG 6.1 standards for safely managed drinking water (1 if yes; 0 if no). We selected all propensity score model covariates *a priori* through a causal diagram informed by the scientific literature to identify potential confounders of the exposure-outcome relationship and additional variables prognostically associated with outcomes.[41] We estimated two propensity score models: (1) a (potential) confounders-only model (community proportions of open defecation and poverty, maternal and paternal education, household wealth quintile, household sanitation, and religion) (S2 Fig); (2) a full model, comprising all potential confounders and possible predictors of child growth outcomes (child age, sex, and birth order, and an interaction between sex and birth order). To ensure that all subjects had a non-zero probability of receiving each treatment[41], we restricted the analysis to the region of common support (individuals for whom the distributions of the propensity scores in treated and controls overlapped). Analyses were restricted to individuals with complete data on all variables; missing data were infrequent. [Fig 1]

#### Main analysis

To estimate the relationships between receipt of treatment and child growth outcomes, the main analysis implemented inverse probability of treatment (IPTW) weighting using the propensity score. We created two synthetic datasets by reweighting subjects by the inverse probability of receiving drinking water meeting SDG 6 standards for “safely managed” water according to our two propensity score models. Prior to estimating the exposure-outcome relationship, we verified that sample balance had been achieved in each of the reweighted synthetic populations, by comparing the distribution of baseline covariates between the treatment groups using standardized differences and by checking for extreme values in the variance ratios by treatment groups.[41] Small standardized differences (rule of thumb, 10% or less) indicate that all important differences in means (measured in units of the pooled standard deviation) of measured baseline covariates have been eliminated by weighting.[41] We then estimated the relationships between the exposure and child growth outcomes of interest in each of the two reweighted samples. We estimated the average treatment effect (“ATE”, defined as the mean of the differences in the outcome between treatment and control groups), and the average treatment effect on the treated (“ATT”, defined as the mean of the differences in the outcome between treatment and control groups among those who received treatment). To gain insights into the determinants of treatment, we predicted potential outcome means for treated and control groups. We also calculated relative treatment effects representing the percentage change in the potential outcome means for treated and control groups.

#### Sensitivity analyses

We repeated IPTW analyses using the continuous length-for-age, weight-for-age, and weight-for-height Z-scores as outcomes. To explore the potential influence of large or influential weights, we re-ran the IPTW analysis truncating for 1% and 5% of the sample.[42] We also ran additional analyses implementing the same two propensity score models using two alternative techniques: propensity score matching[43], and regression adjustment in the IPTW sample, a double-robust method.[44] Propensity score analyses used the “teffects” suite of commands in Stata 15 implemented with robust standard errors. For comparison, we implemented regression models using Generalized Estimating Equations (GEE) to deal with correlations induced by sampling; models otherwise used identical functional forms and variables to the propensity score models.

## Results

### Descriptive and outcome data

Of the 1088 households included in the analysis [Fig 1], 442 (40.62%) received drinking water meeting SDG 6 standards for “safely managed” drinking water, and 646 (59.38%) received drinking water from an improved source that failed to meet SDG 6 standards due to faecal contamination (approximating a “basic” water service level). [Table 1] By contrast to the high water service levels (which reflect the top two rungs on the drinking water ladder), 89.5% (974 of 1088) of households in our sample practiced open defecation daily, representing the lowest rung on the sanitation ladder. We successfully adjusted for systematic baseline differences between households that did and did not receive water meeting SDG 6 “safely managed” drinking water norms using propensity scores. [Tables 1 and S2 and S1 Fig] For the confounders-only model, all absolute standardized differences were less than 1% in the weighted sample, and the largest absolute standardized difference was 0.009. [Table 1] Results for fully adjusted models were similar. [S2 and S3 Tables]

**Table 1:** Baseline characteristics of households drinking "safely managed" water satisfying SDG 6.1 standard compared to households drinking water from an improved source that does not satisfy SDG 6.1 standards, Hardoi district Uttar Pradesh 2013^1^. Confounders-only model.

	SDG 6.1 water^2^ NO (N = 646)	SDG 6.1 water^3^ YES (N = 442)	Standardized difference (absolute)^4^	
			Raw	Weighted
**Outcomes. n (%)**				
Stunting^6^	381 (61.8)	236 (38.3)	—	—
Underweight^7^	388 (62.8)	229 (37.1)	—	—
Wasting^8^	230 (58.8)	161 (41.2)	—	—
**Characteristics of households. parents and children**				
Village proportion poorest, mean (±SD)	0.24 (±0.2)	0.22 (±0.2)	0.089	<0.001
Village proportion open defecation, mean (±SD)	0.86 (±0.2)	0.79 (±0.2)	0.345	0.007
Household wealth quintile, n (%)				
1^st^ quintile (Poorest 20%)	160 (62.9)	94 (37.0)	—	—
2^nd^ quintile	106 (55.2)	86 (44.8)	0.081	0.001
3^rd^ quintile	131 (63.9)	74 (36.1)	0.095	0.009
4^th^ quintile	123 (58.3)	88 (41.7)	0.017	0.009
5^th^ quintile (Richest 20%)	126 (55.8)	100 (44.3)	0.082	0.001
Improved sanitation. n (%)	64 (50.8)	62 (49.2)	0.133	<0.001
Muslim, n (%)	58 (58.6)	41 (41.4)	0.011	0.008
Mother's age (in years), mean (±SD)	28.0 (±5.6)	27.3 (±5.1)	—	—
Maternal education (years), n (%)				
None (0)	389 (62.4)	234 (37.6)	—	—
Some primary (1 to 5)	44 (55.7)	35 (44.3)	0.043	0.001
Some upper primary (6 to 8)	122 (59.5)	83 (40.5)	0.002	0.001
Some secondary or more (≥9)	91 (50.3)	90 (49.7)	0.167	0.002
Paternal education (years), n (%)				
None (0)	186 (60.8)	120 (39.3)	—	—
Some primary (1 to 5)	110 (57.0)	83 (43.0)	0.047	0.007
Some upper primary (6 to 8)	117 (62.6)	70 (37.4)	0.062	<0.001
Some secondary or more (≥9)	233 (58.0)	169 (42.0)	0.041	0.001
Child birth order, n (%)				
1	159 (57.4)	118 (42.6)	—	—
2	147 (60.3)	97 (39.8)	—	—
3	122 (57.6)	90 (42.5)	—	—
4	101 (63.1)	61 (37.7)	—	—
≥5	117 (60.6)	76 (39.4)	—	—
Child sex female, n (%)	310 (57.9)	225 (42.0)	—	—
Child age (in days), mean (±SD)	537.0 (±103.2)	528.0 (±103.4)	—	—

Abbreviations: SDG—Sustainable Development Goals; MDG—Millennium Development Goals; SD—standard deviation

^1^ The analysis sample includes 1088 households, mothers, and children.

^2^ This is drinking water from an ‘improved’ source that fails to meet safety standards for absence of faecal contamination (identified through microbiological testing for E. coli faecal indicator bacteria).

^3^ This is drinking water from an ‘improved’ source that meets safety standards for absence of faecal contamination (identified through microbiological testing for E. coli faecal indicator bacteria).

^4^ These are absolute standardized differences for the confounders-only model

^5^ Asked of the mother of the youngest child 12–23 months in the household. We asked whether she had a child born alive who later died.

^6^ Stunting: length-for-age < –2 standard deviations (SD) of the WHO Child Growth Standards median(36)

^7^ Underweight: weight-for-age < –2 SD of the WHO Child Growth Standards median(36)

^8^ Wasting: weight-for-height < –2 SD of the WHO Child Growth Standards median(36)

### Predictors of treatment group membership

Two variables consistently predicted household drinking water free of faecal contamination (a proxy for the result of successful water treatment, approximating a “safely managed” service level meeting SDG 6.1 norms): a lower proportion of open defecation in the village, and mother in the highest educational category (9^th^ grade or higher). Other variables, including household improved sanitation, were not associated with drinking water quality. [S3 Table] The role of maternal education likely reflects household transmission via hygiene and storage practices as only 8 households reported purifying water.

### Main results

Based on full models, the predicted mean stunting proportion was 58.3% (59.8% underweight, 35.5% wasting) among controls, and 54.8% (52.5% underweight, 36.4% wasting) among the intervention group. [S3 Table] and Table 2 presents the ATE estimates (mean absolute risk differences (RD)) and their 95% confidence intervals. There was no difference in the proportions of stunting or wasting among the treatment and control groups. Treatment decreased the proportion of underweight children in confounders-only (RD -0.074, (95% CI: -0.135 to -0.014; p = 0.017) and full (RD -0.074, (95% CI: -0.134 to -0.013; p = 0.017) models. ATT estimates were similar to those for the general population suggesting that the treatment assignment mechanism was close to random in the original sample. [S4 Table]

**Table 2:** Average treatment effect on selected child health indicators for households drinking water meeting SDG 6.1 norms as compared to those drinking water from an improved source that does not satisfy SDG 6.1 norms, inverse probability of treatment weighted sample (N = 1088).

	Models including only confounders					Full models				
	Average Treatment Effect (ATE)					Average Treatment Effect (ATE)				
Outcomes	Coef.*	Std. Error	95% CI		p-value	Coef.*	Std. Error	95% CI		p-value
**Stunting**	-0.040	0.031	(-0.100;	0.021)	0.197	-0.035	0.031	(-0.095;	0.023)	0.256
**Underweight**	-0.074	0.031	(-0.135;	-0.014)	0.017	-0.074	0.031	(-0.134;	-0.013)	0.017
**Wasting**	0.005	0.030	(-0.053;	0.063)	0.865	0.009	0.030	(-0.049;	0.068)	0.754

*This is the mean absolute risk difference between treatment groups

S5 Table describes anticipated differences in child growth outcomes due to a population-level shift to consumption of safer water meeting SDG 6.1 standards. Based on full models, the average proportion of child underweight is expected to fall by an estimated 12.3% (95% CI: -21.9% to -2.7%) when every household receives water meeting SDG 6 standards for “safely managed” drinking water relative to the case where all households drink water from an improved source that does not satisfy SDG 6 water quality standards.

### Sensitivity analyses

For continuous Z-score nutritional outcomes [S6 Table], fully adjusted models projected non-significant increases among those receiving “safely managed” drinking water meeting SDG 6 standards in mean length-for-age Z score (0.17, (95% CI: -0.01 to 0.35; p = 0.058) and weight-for-age Z score (0.14, (95% CI: -0.00 to 0.28), p = 0.055), and a small non-significant increase in the weight-for-length Z score (0.06 (95% CI: -0.11 to 0.24, p = 0.487). The confidence interval for the weight-for-length Z score was wide. We implemented models for binary outcomes using a range of alternative regression and propensity score techniques; the direction and magnitude of the effects was consistent in models for underweight and stunting. [Table 3] Maximum and minimum weights associated with IPTW models were small, suggesting that the treatment-selection process in the original sample was weak.

**Table 3:** Sensitivity analysis. Estimated effect on selected child growth indicators of households drinking water meeting SDG 6.1 norms as compared to those drinking water from an improved source that does not satisfy SDG 6.1 norms under diverse assumptions, Hardoi district Uttar Pradesh 2013^1^ (N = 1088).

			Stunting			Underweight			Wasting		
	Estimated Weight		Estimated effect			Estimated effect			Estimated effect		
Method	Mean	(Min; Max)	Mean	95% CI		Mean	95% CI		Mean	95% CI	
Logistic regression (unadjusted)^1^	—	—	-0.227	(-0.471;	0.017)	-0.336	(-0.580;	-0.092)	0.036	(-0.216;	0.288)
GEE regression (full models)^2^			-0.149	(-0.441;	0.143)	-0.306	(-0.561;	-0.050)	0.052	(-0.182;	0.226)
PS Matching (full models)			-0.032	(-0.100;	0.035)	-0.067	(-0.139;	0.005)	-0.009	(-0.060;	0.077)
IPTW (full models)^3^	2.002	(1.30; 4.44)	-0.035	(-0.095;	0.023)	-0.074	(-0.134;	-0.013)	0.009	(-0.049;	0.068)
IPTW 1% truncated	2.000	(1.34; 3.86)	-0.035	(-0.097;	0.027)	-0.074	(-0.136;	-0.013)	0.007	(-0.052;	0.067)
IPTW 5% truncated	1.995	(1.40; 3.27)	-0.037	(-0.098;	0.025)	-0.074	(-0.136;	-0.013)	0.008	(-0.052;	0.067)
IPWRA (full models)			-0.036	(-0.095;	0.024)	-0.073	(-0.133;	-0.013)	0.009	(-0.050;	0.067)

Abbreviations: GEE–Generalised Estimating Equations; PS- propensity score; IPTW–inverse-probability of treatment weighted; IPWRA–inverse-probability weighted regression adjustment

^1^ The effect estimate is the difference in the log-odds of the outcome among an average household receiving SDG 6.1 water as compared to households without SDG 6.1 water.

^2^ We implemented GEE models specifying the binomial family with a logit link function, robust standard errors, and an exchangeable correlation structure. The effect estimate is the difference in log-odds of the outcome among an average household receiving SDG 6.1 water as compared to an average household without SDG 6.1 water.

^3^ IPTW coefficients are average treatment effects and represent our main analysis.

## Discussion

We used propensity score techniques to emulate a randomised trial studying whether reduced exposure to faecally contaminated drinking water improves childhood growth in a poor rural Indian locale. The analysis investigates the value of shifting from a “basic” to a “safely managed” water standard by improving water quality, in a setting of very poor sanitation and widespread poverty. Findings showed that children of rural Indian households receiving “safely managed” drinking water were less likely to be underweight, as compared to children from similar households drinking water from an improved source that did not satisfy SDG 6 water quality standards. Evidence concerning the relationship of drinking water meeting SDG 6 water quality norms to length-for-age and weight-for-age was inconclusive, and there was no apparent relationship with stunting or wasting. Based on the magnitude of the coefficient, the most important predictor of water free of faecal contamination was a lower proportion of community open defecation, followed by maternal education of 9^th^ grade of higher.

The WASH Benefits trials reported null findings for the impact of household water treatment on underweight, stunting, wasting, and their associated Z-scores [11, 12]; yet, the applicability of randomised trial findings to new settings and populations is often unclear. We conducted this study to better understand the potential value of water quality improvements for early childhood growth in a specific locale with a distinct configuration of WaSH risks. Similar to the WASH Benefits results, we saw no effect of lower faecal contamination in drinking water on stunting or wasting. However, our finding that the proportion of underweight children in rural Indian households receiving “safely managed” drinking water meeting SDG 6 standards is expected to be lower than in similar households drinking faecally contaminated water from an improved source stands in contrast to the results of the WASH Benefits trials.[11, 12] This divergence may reflect substantially higher background levels of pathogen transmission in our setting than in the Kenyan and especially the Bangladeshi trial sites, representing a higher potential fraction of disease avertable by water treatment.[45] Previous studies of point-of-use interventions to reduce household drinking water contamination, including a well-conducted study by the WASH Benefits investigators in Bangladesh itself,[46] collectively suggest a benefit of these interventions in reducing diarrhoea.[27] Our finding of an anticipated reduction in child underweight likely reflects reduced burdens of infections, diarrhoeal diseases, and environmental enteropathy associated with reduced microbial contamination. While these factors are also relevant for stunting, the aetiology of stunting is particularly complex, change occurs slowly, and dietary, maternal and in-utero factors are important determinants of linear growth.[1, 47] We observed that safer water was possibly associated with length-for-age,[21] but the impact was not large enough to modify clinical stunting in this sample. The aetiology of wasting reflects grave acute causes including severe infectious diseases in early childhood.[1] Although waterborne pathogens may be implicated in some instances, ingestion of water contaminated with *E*. *coli* is unlikely to be a good proxy on aggregate for these severe and relatively rare diseases.

Study strengths include a dataset containing a measure of microbial water quality, which permits us to emulate the effects of a simple water treatment intervention similar to that studied in the WASH Benefits trial (chlorination for household drinking water treatment). Analyses implemented propensity score methods, an appropriate statistical technique to adjust for potential confounding and to mimic the effect of a randomised trial in a new locale with higher levels of environmental contamination than the original WASH Benefits trial sites. Definitions of water and sanitation variables are meaningful for policy as they reflect the new UN SDG service levels. Causal diagrams were used to clarify relationships among variables and design modelling strategies. Findings were robust across a wide range of statistical modelling approaches.

The following limitations should also be considered: (1) Our approximation of the UN JMP WASH ladders for the SDG period is imperfect in two respects. First, to reflect concerns for equity in the burden of water collection, JMP definitions stipulate that total water collection time be less than 30 minutes round trip.[10] Water is abundant and access convenient throughout our study setting; however, water collection time was unavailable in our dataset. Some households might have a “limited” water service level. This change in counterfactual is unlikely to change the anticipated value of water quality improvements.[30] Second, the indicator used to monitor the SDG 6 water quality standard is absence of faecal contamination in drinking water (no *E*. *coli* in 100 mL) based on microbiological testing.[33] By contrast, we assessed faecal contamination in drinking water using the Tara Aquacheck, a hydrogen sulfide bacteria test that correlates well with direct measures of *E*.*coli*.[40] A validation study of the Tara Aquacheck indicates excellent performance (91% true results at 48 hours) but some non-differential misclassification.[40] Although we know that non-differential measurement error in the exposure variable tends to bias results towards the null, we are unable to quantify precisely the impact of possible misclassification on our study results. Moreover, *E*.*coli* is itself simply an indicator bacterium likely to be correlated with the presence of harmful bacteria, viruses and protozoa.[48] (2) Water quality was assessed at a single time point. The study was fielded during the hot and dry season; contamination prevalence is likely to be higher during the rainy period and lower during the winter. Water contamination on a single day is an imperfect proxy for cumulative exposure to contaminated water, likely to lead to non-differential misclassification and attenuation of the effect size. Although variation over time and season is likely, the background factors linked to contamination in our dataset (community open defecation, maternal education) are likely to contribute to stable patterns. (3) Causal inference using the propensity score requires that there be no unmeasured confounders. There is increasing evidence that water contamination may be partly caused by domestic animals.[49] The minimum adjustment model specified in our causal diagram (S2 Fig) required assessment of exposure to domestic animals, but this information was unavailable in our dataset. Exposure to domestic animals and livestock is ubiquitous in this agricultural study setting and unlikely to be substantially imbalanced between treatment groups. (4) We cannot eliminate the possibility of residual confounding as multiple factors contribute to growth faltering, many of which are poorly understood.

Globally, our findings suggest that, in contexts characterised by high pathogen transmission, water quality improvements have the potential to reduce the proportion of underweight children, but may not have strong effects on linear growth, stunting, or wasting. While the international community has emphasised the importance of stunting for purposes of population-level nutrition monitoring, healthy weight gain is also critical for child health.[50]

Two sets of factors may threaten realisation of these modelled benefits in real world settings. (1) Point-of-use water treatment interventions are subject to contamination following treatment through improper storage and handling, and require high adherence.[30] (2) A distinct concern relates to the complexity of pathogen transmission pathways. Previous model-based research has shown that the benefits of water treatment depend on background sanitation and hygiene conditions.[45] Open defecation contributes to circulating pathogens and community open defecation prevalence is closely associated with linear growth failure in rural India.[20] When community-level pathogen transmission is high, water quality improvements may have limited impact irrespective of the amount of water contamination.[45] The WASH Benefits interventions addressed household but not community sanitation and, due to an ensemble of factors, may not have been sufficiently able to alter circulating pathogen levels.[11, 13, 14, 51] Similar factors may be responsible for null results seen in a well-designed point-of-use water treatment trial in Orissa, India.[52] Collectively, they suggest that sustained population-level benefits are more likely to occur through a piped continuous water supply system including centralised treatment as envisaged by SDG 6, but feasibility and cost present important challenges.[14, 30]

## Conclusions

The 2016–2030 Global strategy for women’s, children’s and adolescent’s health aims to ensure that children not only survive, but also that they reach their full growth and development potential.[53] Techniques for causal inference based on observational data can be used to extend the results of randomised trials to new settings, highlighting areas where WASH interventions address a substantial disease burden and have scope for impact. Our results suggest that, in settings of high background pathogen transmission, simple water quality improvements that reduce faecal contamination do have the potential to lower the proportion of underweight children, but are not likely to be associated with linear growth improvements or reduced wasting.

Whether and how water treatment interventions can block environmental contamination sufficiently to make a difference for real-world child health outcomes in high-burden contexts is an urgent research priority. Future studies should investigate point-of-use and infrastructure-based strategies to ensure provision of safe drinking water, alone and in conjunction with comprehensive efforts to reduce circulating pathogen transmission. Innovations in water and sanitation infrastructure adapted to rural settings may be required.[54]

## Supporting information

S1 Table Characteristics of the study setting. (DOCX)

S2 Table Baseline characteristics of households drinking "safely managed" water satisfying SDG 6.1 standard compared to households drinking water from an improved source that does not satisfy SDG 6.1 standards, Hardoi district Uttar Pradesh 2013^1^. Full models.
(DOCX)

S3 Table Potential outcomes means and treatment-model prediction equations. Full models.
(DOCX)

S4 Table Average treatment effect on the treated for selected child health indicators among households drinking water meeting SDG 6.1 norms as compared to those drinking water from an improved source that does not satisfy SDG 6.1 norms, inverse probability of treatment weighted sample (N = 1088). (DOCX)

S5 Table Expected differences in child health outcomes due to a population-level shift to consumption of safer water meeting SDG 6.1 standards. (DOCX)

S6 Table Average treatment effect on continuous Z-score forms of selected child health indicators for households drinking water meeting SDG 6.1 norms as compared to those drinking water from an improved source that does not satisfy SDG 6.1 norms, inverse probability of treatment weighted sample (N = 1088). (DOCX)

S1 Fig Predicted probability of household drinking water meeting SDG 6.1 standards, by treatment group. (DOCX)

S2 Fig Directed Acyclic Graph (Confounders-only model). (DOCX)

S1 Methods Variables and causal pathways included in the Directed Acyclic Graph (DAG). (DOCX)

S1 File Dataset. Analysis dataset.
(CSV)

S2 File Analysis script. Stata code used for analysis.
(DOCX)

S3 File Questionnaire Hindi. Questionnaire used for data collection in Hindi.
(PDF)

S4 File Questionnaire English. English translation of questionnaire used for data collection.
(PDF)

S5 File STROBE checklist. Completed STROBE checklist for an observational cross-sectional study.
(DOC)

## References

[1] Black RE, Victora C, Walker SP, Bhutta ZA, Christian P, De Onis M, et al Maternal and child undernutrition and overweight in low-income and middle-income countries. The Lancet. 2013;382(9890):427–51. 10.1016/S0140-6736(13)60937-X 23746772

[2] United Nations Children's Fund, World Health Organization, World Bank Group. Levels and trends in child malnutrition: Key findings of the 2018 Edition of the Joint Child Malnutrition Estimates 2018 [Available from: https://data.unicef.org/resources/jme.

[3] United Nations. Sustainable Development Knowledge Platform: Sustainable Development Goal 2 2017 [Available from: https://sustainabledevelopment.un.org/sdg2.

[4] Victora CG, Adair L, Fall C, Hallal PC, Martorell R, Richter L, et al Maternal and child undernutrition: consequences for adult health and human capital. Lancet. 2008;371(9609):340–57. 10.1016/S0140-6736(07)61692-4 18206223 PMC2258311

[5] Sudfeld CR, Charles McCoy D, Danaei G, Fink G, Ezzati M, Andrews KG, et al Linear Growth and Child Development in Low- and Middle-Income Countries: A Meta-Analysis. Pediatrics. 2015;135(5):e1266–e75. 10.1542/peds.2014-3111 25847806

[6] Prendergast A, Kelly P. Enteropathies in the developing world: neglected effects on global health. Am J Trop Med Hyg. 2012;86(5):756–63. 10.4269/ajtmh.2012.11-0743 22556071 PMC3335677

[7] Humphrey JH. Child undernutrition, tropical enteropathy, toilets, and handwashing. The Lancet. 2009;374(9694):1032–5. 10.1016/S0140-6736(09)60950-8 19766883

[8] Prendergast AJ, Rukobo S, Chasekwa B, Mutasa K, Ntozini R, Mbuya MN, et al Stunting is characterized by chronic inflammation in Zimbabwean infants. PLoS One. 2014;9(2):e86928 10.1371/journal.pone.0086928 24558364 PMC3928146

[9] Sanitation Hygiene Infant Nutrition Efficacy Trial T, Humphrey JH, Jones AD, Manges A, Mangwadu G, Maluccio JA, et al The Sanitation Hygiene Infant Nutrition Efficacy (SHINE) Trial: Rationale, Design, and Methods. Clin Infect Dis. 2015;61 Suppl 7:S685–702. 26602296 10.1093/cid/civ844 PMC4657589

[10] WHO/ UNICEF Joint Monitoring Program on Water and Sanitation (JMP). Progress on drinking water, sanitation and hygiene 2017 update and SDG baselines. Geneva, Switzerland: World Health Organization; 2017.

[11] Luby SP, Rahman M, Arnold BF, Unicomb L, Ashraf S, Winch PJ, et al Effects of water quality, sanitation, handwashing, and nutritional interventions on diarrhoea and child growth in rural Bangladesh: a cluster randomised controlled trial. Lancet Glob Health. 2018;6(3):e302–e15. 10.1016/S2214-109X(17)30490-4 29396217 PMC5809718

[12] Null C, Stewart CP, Pickering AJ, Dentz HN, Arnold BF, Arnold CD, et al Effects of water quality, sanitation, handwashing, and nutritional interventions on diarrhoea and child growth in rural Kenya: a cluster-randomised controlled trial. Lancet Glob Health. 2018;6(3):e316–e29. 10.1016/S2214-109X(18)30005-6 29396219 PMC5809717

[13] Coffey D, Spears D. Implications of WASH Benefits trials for water and sanitation. Lancet Glob Health. 2018;6(6):e615 10.1016/S2214-109X(18)30225-0 29706564

[14] Cumming O, Curtis V. Implications of WASH Benefits trials for water and sanitation. Lancet Glob Health. 2018;6(6):e613–e4. 10.1016/S2214-109X(18)30192-X 29706563

[15] United Nations. UN-Water: Coordinating the UN's work on water and sanitation—Integrated Monitoring Initiative for SDG 6 2017 [Available from: http://www.sdg6monitoring.org/.

[16] Arnold BF, Null C, Luby SP, Colford JM Jr. Implications of WASH Benefits trials for water and sanitation—Authors' reply. Lancet Glob Health. 2018;6(6):e616–e7. 10.1016/S2214-109X(18)30229-8 29706562

[17] Johri M, Chandra D, Subramanian SV, Sylvestre MP, Pahwa S. MDG 7c for safe drinking water in India: an illusive achievement. Lancet. 2014;383(9926):1379. 10.1016/S0140-6736(14)60673-5 24759242

[18] Johri M, Subramanian SV, Kone GK, Dudeja S, Chandra D, Minoyan N, et al Maternal Health Literacy Is Associated with Early Childhood Nutritional Status in India. J Nutr. 2016;146(7):1402–10. 10.3945/jn.115.226290 27306895

[19] India State-Level Disease Burden Initiative C. Nations within a nation: variations in epidemiological transition across the states of India, 1990–2016 in the Global Burden of Disease Study. Lancet. 2017;390(10111):2437–60. 10.1016/S0140-6736(17)32804-0 29150201 PMC5720596

[20] Spears D. How much international variation in child height can sanitation explain? Policy research working paper; no. WPS 6351. Washington, DC, USA: The World Bank; 2013 2013/01/01.

[21] Dangour AD, Watson L, Cumming O, Boisson S, Che Y, Velleman Y, et al Interventions to improve water quality and supply, sanitation and hygiene practices, and their effects on the nutritional status of children. Cochrane Database Syst Rev. 2013;8:Cd009382. 10.1002/14651858.CD009382.pub2 PMC11608819 23904195

[22] Fink G, Gunther I, Hill K. The effect of water and sanitation on child health: evidence from the demographic and health surveys 1986–2007. Int J Epidemiol. 2011;40(5):1196–204. 10.1093/ije/dyr102 21724576

[23] Pickering AJ, Djebbari H, Lopez C, Coulibaly M, Alzua ML. Effect of a community-led sanitation intervention on child diarrhoea and child growth in rural Mali: a cluster-randomised controlled trial. Lancet Glob Health. 2015;3(11):e701–11. 10.1016/S2214-109X(15)00144-8 26475017

[24] Clasen T, Boisson S, Routray P, Torondel B, Bell M, Cumming O, et al Effectiveness of a rural sanitation programme on diarrhoea, soil-transmitted helminth infection, and child malnutrition in Odisha, India: a cluster-randomised trial. Lancet Glob Health. 2014;2(11):e645–53. 10.1016/S2214-109X(14)70307-9 25442689

[25] Patil SR, Arnold BF, Salvatore AL, Briceno B, Ganguly S, Colford JM Jr., et al The effect of India's total sanitation campaign on defecation behaviors and child health in rural Madhya Pradesh: a cluster randomized controlled trial. PLoS Med. 2014;11(8):e1001709 10.1371/journal.pmed.1001709 25157929 PMC4144850

[26] Coffey D, Spears D. Where India Goes: Abandoned Toilets, Stunted Development, and the Costs of Caste: HarperCollins; 2017.

[27] Clasen TF, Alexander KT, Sinclair D, Boisson S, Peletz R, Chang HH, et al Interventions to improve water quality for preventing diarrhoea. Cochrane Database Syst Rev. 2015;10:CD004794. 10.1002/14651858.CD004794.pub3 PMC4625648 26488938

[28] Johri M, Subramanian SV, Sylvestre MP, Dudeja S, Chandra D, Kone GK, et al Association between maternal health literacy and child vaccination in India: a cross-sectional study. J Epidemiol Community Health. 2015. 10.1136/jech-2014-205436 PMC4552929 25827469

[29] Freeman MC, Garn JV, Sclar GD, Boisson S, Medlicott K, Alexander KT, et al The impact of sanitation on infectious disease and nutritional status: A systematic review and meta-analysis. International Journal of Hygiene and Environmental Health. 2017;220(6):928–49. 10.1016/j.ijheh.2017.05.007 28602619

[30] Wolf J, Pruss-Ustun A, Cumming O, Bartram J, Bonjour S, Cairncross S, et al Assessing the impact of drinking water and sanitation on diarrhoeal disease in low- and middle-income settings: systematic review and meta-regression. Trop Med Int Health. 2014;19(8):928–42. 10.1111/tmi.12331 24811732

[31] Shields KF, Bain RE, Cronk R, Wright JA, Bartram J. Association of Supply Type with Fecal Contamination of Source Water and Household Stored Drinking Water in Developing Countries: A Bivariate Meta-analysis. Environ Health Perspect. 2015;123(12):1222–31. 10.1289/ehp.1409002 25956006 PMC4671240

[32] Bain R, Cronk R, Wright J, Yang H, Slaymaker T, Bartram J. Fecal contamination of drinking-water in low- and middle-income countries: a systematic review and meta-analysis. PLoS Med. 2014;11(5):e1001644 10.1371/journal.pmed.1001644 24800926 PMC4011876

[33] WHO/ UNICEF. Safely managed drinking water—thematic report on drinking water 2017. Geneva, Switzerland: World Health Organization; 2017.

[34] WHO/ UNICEF. Joint Monitoring Programme (JMP) for Water Supply and Sanitation 2017 “The new JMP ladder for drinking water” https://washdata.org/monitoring/drinking-water Accessed July 22, 2018.

[35] Gilmartin AA, Petri WA Jr. Exploring the role of environmental enteropathy in malnutrition, infant development and oral vaccine response. Philos Trans R Soc Lond B Biol Sci. 2015;370(1671). 10.1098/rstb.2014.0143 PMC4527388 25964455

[36] World Health Organization. WHO child growth standards: length/height-for-age, weight-for-age, weight-for-length, weight-for-height and body mass index-for-age: methods and development. France: WHO Press; 2006.

[37] World Health Organization. WHO Anthro. 3.2.2 "i grow up" macro for Stata ed. Geneva: World Health Organization; 2011.

[38] TARA Enviro. TARA Aquacheck Vial 2013.

[39] WHO / UNICEF. Joint Monitoring Programme (JMP) for Water Supply and Sanitation: Progress on sanitation and drinking water 2013 update2013.

[40] Murcott S, Keegan M, A H, Jain A, Knutson J, Liu S, et al Evaluation of microbial water quality tests for humanitarian emergency and development settings. Procedia Engineering. 2015;107:237–46.

[41] Austin PC, Stuart EA. Moving towards best practice when using inverse probability of treatment weighting (IPTW) using the propensity score to estimate causal treatment effects in observational studies. Stat Med. 2015;34(28):3661–79. 10.1002/sim.6607 26238958 PMC4626409

[42] Cole SR, Hernan MA. Constructing inverse probability weights for marginal structural models. Am J Epidemiol. 2008;168(6):656–64. 10.1093/aje/kwn164 18682488 PMC2732954

[43] Rosenbaum PR, Rubin DB. The central role of the propensity score in observational studies for causal effects. Biometrika. 1983;70(1):41–55.

[44] Imbens GW, Wooldridge JM. Recent Developments in the Econometrics of Program Evaluation. J Econ Lit. 2009;47(1):5–86.

[45] Eisenberg JN, Scott JC, Porco T. Integrating disease control strategies: balancing water sanitation and hygiene interventions to reduce diarrheal disease burden. Am J Public Health. 2007;97(5):846–52. 10.2105/AJPH.2006.086207 17267712 PMC1854876

[46] Ercumen A, Naser AM, Unicomb L, Arnold BF, Colford JM Jr, Luby SP. Effects of Source- versus Household Contamination of Tubewell Water on Child Diarrhea in Rural Bangladesh: A Randomized Controlled Trial. PLOS ONE. 2015;10(3):e0121907 10.1371/journal.pone.0121907 25816342 PMC4376788

[47] Kim R, Mejia-Guevara I, Corsi DJ, Aguayo VM, Subramanian SV. Relative importance of 13 correlates of child stunting in South Asia: Insights from nationally representative data from Afghanistan, Bangladesh, India, Nepal, and Pakistan. Soc Sci Med. 2017;187:144–54. 10.1016/j.socscimed.2017.06.017 28686964

[48] World Health Organization. Guidelines for drinking-water quality2011.

[49] Daniels ME, Shrivastava A, Smith WA, Sahu P, Odagiri M, Misra PR, et al Cryptosporidium and Giardia in Humans, Domestic Animals, and Village Water Sources in Rural India. Am J Trop Med Hyg. 2015;93(3):596–600. 10.4269/ajtmh.15-0111 26123963 PMC4559703

[50] Lutter CK, Chaparro CM, Munoz S. Progress towards Millennium Development Goal 1 in Latin America and the Caribbean: the importance of the choice of indicator for undernutrition. Bull World Health Organ. 2011;89(1):22–30. 10.2471/BLT.10.078618 21346887 PMC3040018

[51] Ercumen A, Pickering AJ, Kwong LH, Mertens A, Arnold BF, Benjamin-Chung J, et al Do Sanitation Improvements Reduce Fecal Contamination of Water, Hands, Food, Soil, and Flies? Evidence from a Cluster-Randomized Controlled Trial in Rural Bangladesh. Environmental Science & Technology. 2018;52(21):12089–97. 10.1021/acs.est.8b02988 PMC6222553 30256095

[52] Boisson S, Stevenson M, Shapiro L, Kumar V, Singh LP, Ward D, et al Effect of household-based drinking water chlorination on diarrhoea among children under five in Orissa, India: a double-blind randomised placebo-controlled trial. PLoS Med. 2013;10(8):e1001497 10.1371/journal.pmed.1001497 23976883 PMC3747993

[53] Every Woman Every Child. Global Strategy for Women's, Children's and Adolescents Health 2016–2030: Survive, Thrive, Transform2015. 108 p.

[54] Duflo E, Greenstone M, Guiteras R, Clasen T. The Short- and Medium-Term Impacts of Household Water Supply and Sanitation on Diarrhea in Rural India. Maryland Population Research Centre; 2015 April 2015. Contract No.: PWP-MPRC-2015-008.

