# Supplementary material for: Effects of improved drinking water quality on early childhood growth in rural Uttar Pradesh, India: A propensity-score analysis
Source: PLoS One. 2019 Jan 8;14(1):e0209054. doi: 10.1371/journal.pone.0209054 (PMC6324831; doi:10.1371/journal.pone.0209054)
Supplement: S1 Table — (DOCX) [file pone.0209054.s001.docx]

### Table S1. Characteristics of the Study Setting

|  | Population  (millions)[1] | Under-5 Mortality  (per 1000)[2, 3] | Nutritional status of children 0-59 months (%)[4, 5] | | |
| --- | --- | --- | --- | --- | --- |
|  |  |  | **Stunting** | **Underweight** | **Wasting** |
| India | 1210 | 57.3 | 38.4 | 35.7 | 21.0 |
| State of Uttar Pradesh (UP) | 200 | 90 | 46.3 | 39.5 | 17.9 |
| Hardoi district, UP | 4 | 118 | 52.9 | 42.1 | 16.2 |

Updated national and state estimates for under-5 mortality are available from the NHFS-4: India: 50 (56 rural); Uttar Pradesh 78 (80 rural).[4, 5] No updated NHFS-4 under-5 mortality estimates are available for districts. The under-5 mortality rate changes rapidly and data collection for this study was done in 2013 prior to the NFHS-4 data collection in 2015-2016; moreover, no Hardoi district estimates are available from the NFHS-4. Table S1 therefore does not present NFHS-4 under-5 mortality estimates.

**References**

1. Office of the Registrar General and Census Commissioner. Population Tables : India : Census 2011. New Delhi: Government of India, Ministry of Home Affairs. , 2014.

2. Office of the Registrar General and Census Commissioner (India). Annual Health Survey : 2012-13 Fact Sheet: Uttar Pradesh. New Delhi: Government of India, Ministry of Home Affairs.

3. Ram U, Jha P, Ram F, Kumar K, Awasthi S, Shet A, et al. Neonatal, 1–59 month, and under-5 mortality in 597 Indian districts, 2001 to 2012: estimates from national demographic and mortality surveys. The Lancet Global Health. 2013;1(4):e219-e26. doi: 10.1016/s2214-109x(13)70073-1.

4. International Institute for Population Sciences (IIPS). National Family Health Survey (NFHS-4), 2015-16: India. Mumbai: IIPS, 2016.

5. International Institute for Population Sciences (IIPS) and ICF. National Family Health Survey (NFHS-4), India, 2015-16: Uttar Pradesh. Mumbai: IIPS, 2017.
