# Supplementary material for: Effects of improved drinking water quality on early childhood growth in rural Uttar Pradesh, India: A propensity-score analysis
Source: PLoS One. 2019 Jan 8;14(1):e0209054. doi: 10.1371/journal.pone.0209054 (PMC6324831; doi:10.1371/journal.pone.0209054)
Supplement: S2 Table — Full models. (DOCX) [file pone.0209054.s002.docx]

### Table S2. Baseline characteristics of households drinking "safely managed" water satisfying SDG 6.1 standard compared to households drinking water from an improved source that does not satisfy SDG 6.1 standards, Hardoi district Uttar Pradesh 2013^1^: full models

|  | | | **SDG 6.1 water^2^ NO**  **(N=646)** | | **SDG 6.1 water^3^ YES**  **(N=442)** | | **Standardized difference (absolute)^4^** | | |
| --- | --- | --- | --- | --- | --- | --- | --- | --- | --- |
|  |  |  |  |  |  |  | **Raw** | **Weighted** | |
| **Outcomes, n (%)** | |  | |  | |  | |  |  |
|  | Stunting^6^ | 381 (61.8) | | 236 (38.3) | | -- | | -- |  |
|  | Underweight^7^ | 388 (62.8) | | 229 (37.1) | | -- | | -- |  |
|  | Wasting^8^ | 230 (58.8) | | 161 (41.2) | | -- | | -- |  |
| **Characteristics of households, parents and children** | |  | |  | |  | | |  |
| Village proportion poorest, mean (±SD) | | 0.24 (±0.2) | | 0.22 (±0.2) | | 0.089 | | 0.002 |  |
| Village proportion open defecation, mean (±SD) | | 0.86 (±0.2) | | 0.79 (±0.2) | | 0.345 | | 0.009 |  |
| Household wealth quintile, n (%) | |  | |  | |  | |  |  |
|  | 1^st^ quintile (Poorest 20%) | 160 (62.9) | | 94 (37.0) | | -- | | -- |  |
|  | 2^nd^ quintile | 106 (55.2) | | 86 (44.8) | | 0.081 | | 0.003 |  |
|  | 3^rd^ quintile | 131 (63.9) | | 74 (36.1) | | 0.095 | | 0.016 |  |
|  | 4^th^ quintile | 123 (58.3) | | 88 (41.7) | | 0.017 | | 0.012 |  |
|  | 5^th^ quintile (Richest 20%) | 126 (55.8) | | 100 (44.3) | | 0.082 | | 0.001 |  |
| Improved sanitation, n (%) | | 64 (50.8) | | 62 (49.2) | | 0.133 | | 0.004 |  |
| Muslim, n (%) | | 58 (58.6) | | 41 (41.4) | | 0.011 | | 0.007 |  |
| Mother's age (in years), mean (±SD) | | 28.0 (±5.6) | | 27.3 (±5.1) | | -- | | -- |  |
| Maternal education (years), n (%) | |  | |  | |  | |  |  |
|  | None (0) | 389 (62.4) | | 234 (37.6) | | -- | | -- |  |
|  | Some primary (1 to 5) | 44 (55.7) | | 35 (44.3) | | 0.043 | | 0.003 |  |
|  | Some upper primary (6 to 8) | 122 (59.5) | | 83 (40.5) | | 0.002 | | 0.000 |  |
|  | Some secondary or more (≥9) | 91 (50.3) | | 90 (49.7) | | 0.167 | | 0.002 |  |
| Paternal education (years), n (%) | |  | |  | |  | |  |  |
|  | None (0) | 186 (60.8) | | 120 (39.3) | | -- | | -- |  |
|  | Some primary (1 to 5) | 110 (57.0) | | 83 (43.0) | | 0.047 | | 0.000 |  |
|  | Some upper primary (6 to 8) | 117 (62.6) | | 70 (37.4) | | 0.062 | | 0.004 |  |
|  | Some secondary or more (≥9) | 233 (58.0) | | 169 (42.0) | | 0.041 | | 0.003 |  |
| Child birth order, n (%) | |  | |  | |  | |  |  |
|  | 1 | 159 (57.4) | | 118 (42.6) | | -- | | -- |  |
|  | 2 | 147 (60.3) | | 97 (39.8) | | 0.018 | | 0.002 |  |
|  | 3 | 122 (57.6) | | 90 (42.5) | | 0.038 | | 0.007 |  |
|  | 4 | 101 (63.1) | | 61 (37.7) | | 0.064 | | 0.002 |  |
|  | ≥5 | 117 (60.6) | | 76 (39.4) | | 0.015 | | 0.004 |  |
| Child sex female, n (%) | | 310 (57.9) | | 225 (42.0) | | 0.056 | | 0.010 |  |
| Interaction (female x birth order) | |  | |  | |  | |  |  |
|  | female x 1 |  | |  | | 0.055 | | 0.003 |  |
|  | female x 2 |  | |  | | 0.022 | | 0.003 |  |
|  | female x 3 |  | |  | | 0.062 | | 0.010 |  |
|  | female x 4 |  | |  | | 0.021 | | 0.008 |  |
|  | female x ≥5 |  | |  | | -- | | -- |  |
| Child age (in days). mean (±SD) | | 537.0 (±103.2) | | 528.0 (±103.4) | |  | |  |  |
|  | Age spline 1 |  | |  | | 0.091 | | 0.004 |  |
|  | Age spline 2 |  | |  | | 0.083 | | 0.005 |  |
|  | Age spline 3 |  | |  | | 0.077 | | 0.005 |  |
|  | Age spline 4 |  | |  | | 0.063 | | 0.004 |  |

Abbreviations: SDG - Sustainable Development Goals; MDG - Millennium Development Goals; SD - standard deviation

^1^ The analysis sample includes 1088 households, mothers, and children.

^2^ This is drinking water from an ‘improved’ source that fails to meet safety standards for absence of faecal contamination (identified through microbiological testing for E. coli faecal indicator bacteria)..

^3^ This is drinking water from an ‘improved’ source that meets safety standards for absence of faecal contamination (identified through microbiological testing for E. coli faecal indicator bacteria).

^4^ These are absolute standardized differences for the nutritional outcomes models

^5^ Asked of the mother of the youngest child 12-23 months in the household. We asked whether she had a child born alive who later died.

^6^ Stunting: length-for-age < –2 standard deviations (SD) of the WHO Child Growth Standards median[1]

^7^ Underweight: weight-for-age < –2 SD of the WHO Child Growth Standards median[1]

^8^ Wasting: weight-for-height < –2 SD of the WHO Child Growth Standards median[1]

**References**

1. World Health Organization. WHO child growth standards : length/height-for-age, weight-for-age, weight-for-length, weight-for-height and body mass index-for-age : methods and development. France: WHO Press; 2006.
