# Supplementary material for: Effects of improved drinking water quality on early childhood growth in rural Uttar Pradesh, India: A propensity-score analysis
Source: PLoS One. 2019 Jan 8;14(1):e0209054. doi: 10.1371/journal.pone.0209054 (PMC6324831; doi:10.1371/journal.pone.0209054)
Supplement: S3 Table — Full models. (DOCX) [file pone.0209054.s003.docx]

### Table S3. Potential outcomes means and treatment-model prediction equations: full models

| **Predicted Outcomes Means (Growth outcomes)^1^** | | | **Coef.*** | **Std. Error** | **95% CI** | | **p-value** |
| --- | --- | --- | --- | --- | --- | --- | --- |
| Stunting | | |  |  |  |  |  |
|  | | SDG water = No | 0.583 | 0.020 | 0.545 | 0.621 | <0.001 |
|  | | SDG water = Yes | 0.548 | 0.024 | 0.502 | 0.595 | <0.001 |
| Underweight | | |  |  |  |  |  |
|  | SDG water = No | | 0.598 | 0.019 | 0.564 | 0.640 | <0.001 |
|  | SDG water = Yes | | 0.525 | 0.024 | 0.478 | 0.572 | <0.001 |
| Wasting | | |  |  |  |  |  |
|  | SDG water = No | | 0.355 | 0.019 | 0.318 | 0.392 | <0.001 |
|  | SDG water = Yes | | 0.364 | 0.023 | 0.319 | 0.409 | <0.001 |
| **Treatment-model prediction equations^2^** | | | | | | | |
| Village proportion poorest, mean (±SD) | | | -0.324 | 0.454 | -1.214 | 0.565 | 0.475 |
| Village proportion open defecation, mean (±SD) | | | -1.476 | 0.308 | -2.079 | -0.873 | <0.001 |
| Household wealth quintile, n (%) | | |  |  |  |  |  |
|  | 1^st^ quintile (Poorest 20%) (Ref.) | | -- | -- | -- | -- | -- |
|  | 2^nd^ quintile | | 0.279 | 0.206 | -0.125 | 0.684 | 0.176 |
|  | 3^rd^ quintile | | -0.159 | 0.216 | -0.582 | 0.263 | 0.460 |
|  | 4^th^ quintile | | 0.087 | 0.206 | -0.316 | 0.491 | 0.671 |
|  | 5^th^ quintile (Richest 20%) | | -0.139 | 0.255 | -0.638 | 0.361 | 0.586 |
| Improved sanitation. n (%) | | | 0.313 | 0.266 | -0.210 | 0.835 | 0.241 |
| Muslim, n (%) | | | 0.054 | 0.218 | -0.373 | 0.481 | 0.804 |
| Girl | | | -0.156 | 0.300 | -0.745 | 0.433 | 0.603 |
| Mother's age (in years), mean (±SD) | | |  |  |  |  |  |
|  | Age spline 1 | | -0.002 | 0.004 | -0.011 | 0.006 | 0.581 |
|  | Age spline 2 | | 0.012 | 0.021 | -0.030 | 0.053 | 0.580 |
|  | Age spline 3 | | -0.045 | 0.067 | -0.177 | 0.086 | 0.499 |
|  | Age spline 4 | | 0.101 | 0.122 | -0.137 | 0.339 | 0.406 |
|  | Age spline 5 (Ref.) | | -- | -- | -- | -- | -- |
| Maternal education (years), n (%) | | |  |  |  |  |  |
|  | None (0) (Ref.) | | -- | -- | -- | -- | -- |
|  | Some primary (1 to 5) | | 0.104 | 0.263 | -0.411 | 0.620 | 0.692 |
|  | Some upper primary (6 to 8) | | 0.137 | 0.175 | -0.206 | 0.479 | 0.435 |
|  | Some secondary or more (≥9) | | 0.455 | 0.192 | 0.079 | 0.832 | 0.018 |
| Paternal education (years), n (%) | | |  |  |  |  |  |
|  | None (0) (Ref.) | | -- | -- | -- | -- | -- |
|  | Some primary (1 to 5) | | 0.089 | 0.206 | -0.314 | 0.492 | 0.663 |
|  | Some upper primary (6 to 8) | | -0.072 | 0.199 | -0.462 | 0.318 | 0.716 |
|  | Some secondary or more (≥9) | | -0.006 | 0.171 | -0.341 | 0.330 | 0.974 |
| Birth order | | |  |  |  |  |  |
|  | 1 (Ref.) | | -- | -- | -- | -- | -- |
|  | 2 | | -0.244 | 0.268 | -0.769 | 0.281 | 0.363 |
|  | 3 | | -0.004 | 0.274 | -0.541 | 0.534 | 0.989 |
|  | 4 | | -0.174 | 0.300 | -0.763 | 0.415 | 0.562 |
|  | 5 or more | | 0.086 | 0.276 | -0.456 | 0.628 | 0.756 |
| Girl x Birth order | | |  |  |  |  |  |
|  | 1*1 | | 0.306 | 0.393 | -0.463 | 1.076 | 0.435 |
|  | 1*2 | | 0.438 | 0.405 | -0.357 | 1.233 | 0.280 |
|  | 1*3 | | 0.368 | 0.416 | -0.448 | 1.183 | 0.377 |
|  | 1*4 | | 0.366 | 0.453 | -0.521 | 1.253 | 0.418 |
|  | 1*5 or more (Ref.) | | -- | -- | -- | -- | -- |

^1^ Coefficients represent the predicted proportion of the sample with the outcome, by treatment group

^2^ These are the potential predictors of households receiving water meeting SDG 6.1 norms
