# Supplementary material for: Effects of improved drinking water quality on early childhood growth in rural Uttar Pradesh, India: A propensity-score analysis
Source: PLoS One. 2019 Jan 8;14(1):e0209054. doi: 10.1371/journal.pone.0209054 (PMC6324831; doi:10.1371/journal.pone.0209054)
Supplement: S4 Table — (DOCX) [file pone.0209054.s004.docx]

### Table S4. Average treatment effect on the treated for selected child health indicators among households drinking water meeting SDG 6.1 norms as compared to those drinking water from an improved source that does not satisfy SDG 6.1 norms, inverse probability of treatment weighted sample (N=1088)

|  | **Models including only confounders** | | | | | **Full models** | | | | |
| --- | --- | --- | --- | --- | --- | --- | --- | --- | --- | --- |
|  | **Average Treatment on the Treated (ATT)** | | | | | **Average Treatment on the Treated (ATT)** | | | | |
| **Outcomes** | **Coef.*** | **Std. Error** | **95% CI** | | **p-value** | **Coef.*** | **Std. Error** | **95% CI** | | **p-value** |
| **Stunting** | -0.045 | 0.032 | (-0.109; | 0.018) | 0.160 | -0.039 | 0.032 | (-0.102; | 0.024) | 0.226 |
| **Underweight** | -0.078 | 0.032 | (-0.140; | -0.015) | 0.015 | -0.077 | 0.032 | (-0.139; | -0.015) | 0.016 |
| **Wasting** | 0.009 | 0.031 | (-0.051; | 0.070) | 0.766 | 0.010 | 0.031 | (-0.050; | 0.071) | 0.735 |

*This is the mean absolute risk difference between treatment groups
