# Supplementary material for: Effects of improved drinking water quality on early childhood growth in rural Uttar Pradesh, India: A propensity-score analysis
Source: PLoS One. 2019 Jan 8;14(1):e0209054. doi: 10.1371/journal.pone.0209054 (PMC6324831; doi:10.1371/journal.pone.0209054)
Supplement: S5 Table — (DOCX) [file pone.0209054.s005.docx]

### Table S5. Expected differences in child health outcomes due to a population-level shift to consumption of safer water meeting SDG 6.1 standards

|  | Propensity Score Weighting (IPTW)  Models with only confounders | Propensity Score Weighting (IPTW)  Full models |
| --- | --- | --- |
| Stunting  (-2SD) | The average proportion of child stunting falls by an estimated 6.8% when every household received water meeting SDG 6.1 standards relative to the case when no households receive SDG 6.1 water (but all receive basic water meeting MDG 7c standards). We also obtain a 95% confidence interval of a 16.9% reduction to 3.2% increase. | The average proportion of child stunting falls by an estimated 6.0% when every household received water meeting SDG 6.1 standards relative to the case when no households receive SDG 6.1 water (but all receive basic water meeting MDG 7c standards). We also obtain a 95% confidence interval of a 15.9% reduction to 4.1% increase. |
| Underweight  (-2SD) | The average proportion of child underweight falls by an estimated 12.4% when every household received water meeting SDG 6.1 standards relative to the case when no households receive SDG 6.1 water (but all receive basic water meeting MDG 7c standards). We also obtain a 95% confidence interval of a 22.0% to 2.7% reduction. | The average proportion of child underweight falls by an estimated 12.3% when every household received water meeting SDG 6.1 standards relative to the case when no households receive SDG 6.1 water (but all receive basic water meeting MDG 7c standards). We also obtain a 95% confidence interval of a 21.9% to a 2.7% reduction. |
| Wasting  (-2SD) | The average proportion of child wasting increases by an estimated 1.4% when every household received water meeting SDG 6.1 standards relative to the case when no households receive SDG 6.1 water (but all receive basic water meeting MDG 7c standards). We also obtain a 95% confidence interval of a 15.1% reduction to 17.9% increase. | The average proportion of child wasting falls by an estimated 2.6% when every household received water meeting SDG 6.1 standards relative to the case when no households receive SDG 6.1 water (but all receive basic water meeting MDG 7c standards). We also obtain a 95% confidence interval of a 13.9% reduction to 19.2% increase. |
