# Supplementary material for: Effects of improved drinking water quality on early childhood growth in rural Uttar Pradesh, India: A propensity-score analysis
Source: PLoS One. 2019 Jan 8;14(1):e0209054. doi: 10.1371/journal.pone.0209054 (PMC6324831; doi:10.1371/journal.pone.0209054)
Supplement: S6 Table — (DOCX) [file pone.0209054.s006.docx]

### Table S6. Average treatment effect on continuous Z-score forms of selected child health indicators for households drinking water meeting SDG 6.1 norms as compared to those drinking water from an improved source that does not satisfy SDG 6.1 norms, inverse probability of treatment weighted sample (N=1088)

|  | **Models including only confounders** | | | | | **Full models** | | | | |
| --- | --- | --- | --- | --- | --- | --- | --- | --- | --- | --- |
|  | **Average Treatment Effect (ATE)** | | | | | **Average Treatment Effect (ATE)** | | | | |
| **Outcomes^1^** | **Coef.*** | **Std. Error** | **95% CI** | | **p-value** | **Coef.*** | **Std. Error** | **95% CI** | | **p-value** |
| **Height-for-age**  **(HAZ)** | 0.185 | 0.094 | (0.001; | 0.368) | 0.049 | 0.174 | 0.092 | (-0.006; | 0.354) | 0.058 |
| **Weight-for-age**  **(WAZ)** | 0.147 | 0.073 | (0.005; | 0.289) | 0.043 | 0.138 | 0.072 | (-0.003; | 0.279) | 0.055 |
| **Weight-for-height**  **(WHZ)** | 0.071 | 0.089 | (-0.102; | 0.245) | 0.420 | 0.061 | 0.088 | (-0.112; | 0.235) | 0.487 |

*This is the mean absolute risk difference between treatment groups

1. Height-for-age *Z*-scores (HAZ), weight-for-age *Z*-scores (WAZ) and weight-for-height *Z*-scores (WHZ) calculated with reference to the WHO Multicentre Growth Reference Study population.[1] Standardized z-scores have mean 0 and a standard deviation of 1.

**References**

1. World Health Organization. WHO child growth standards : length/height-for-age, weight-for-age, weight-for-length, weight-for-height and body mass index-for-age : methods and development. France: WHO Press; 2006.
