# Supplementary material for: Effects of improved drinking water quality on early childhood growth in rural Uttar Pradesh, India: A propensity-score analysis
Source: PLoS One. 2019 Jan 8;14(1):e0209054. doi: 10.1371/journal.pone.0209054 (PMC6324831; doi:10.1371/journal.pone.0209054)
Supplement: S1 Fig — (DOCX) [file pone.0209054.s007.docx]

### Figure S1. Predicted probability of household drinking water meeting SDG 6.1 standards, by treatment group


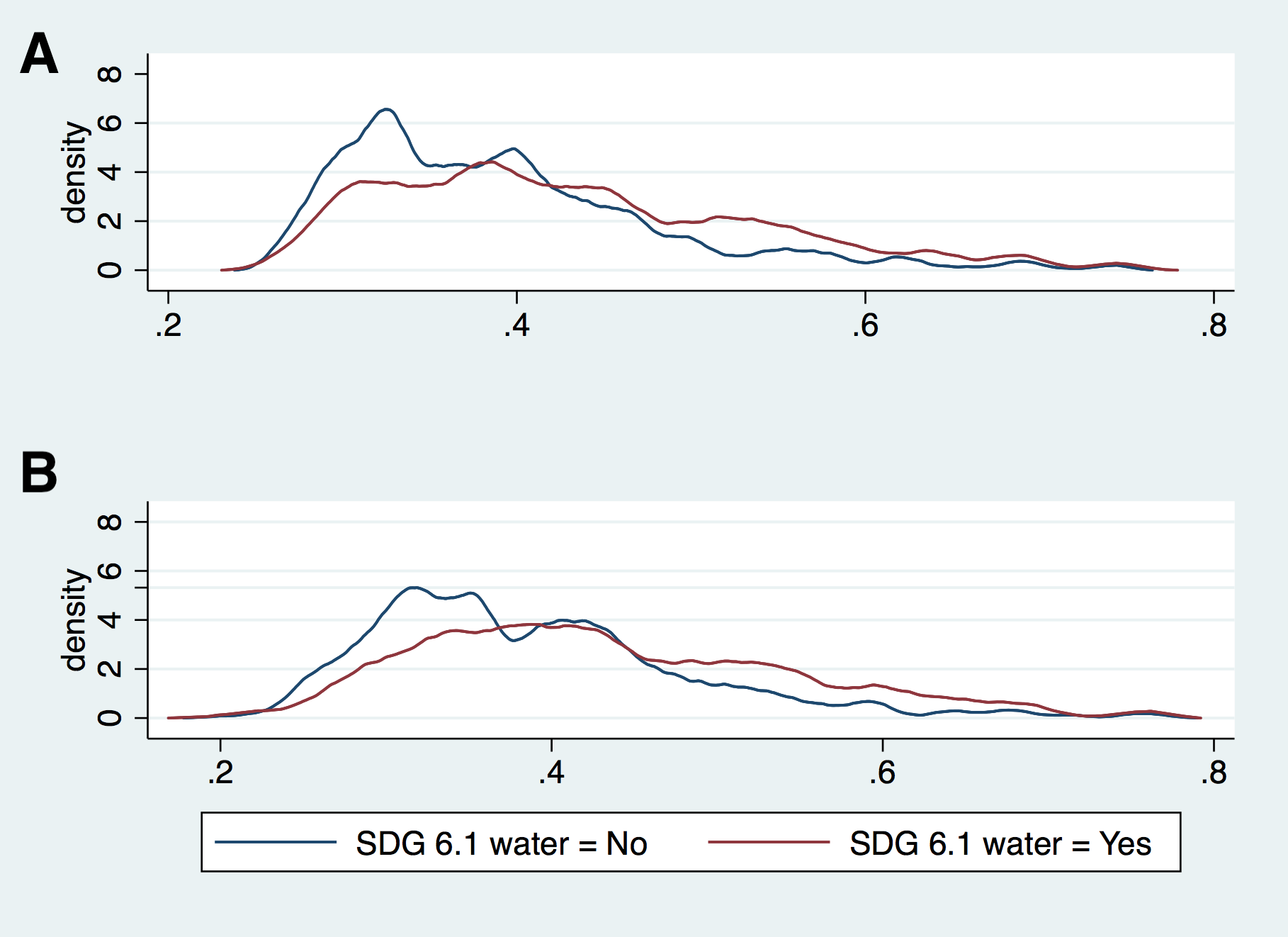


A: Confounders-only model; B: Full models
