# Supplementary material for: Effects of improved drinking water quality on early childhood growth in rural Uttar Pradesh, India: A propensity-score analysis
Source: PLoS One. 2019 Jan 8;14(1):e0209054. doi: 10.1371/journal.pone.0209054 (PMC6324831; doi:10.1371/journal.pone.0209054)
Supplement: S2 Fig — (DOCX) [file pone.0209054.s008.docx]

### Figure S2. - Directed Acyclic Graph (Confounders-only model)


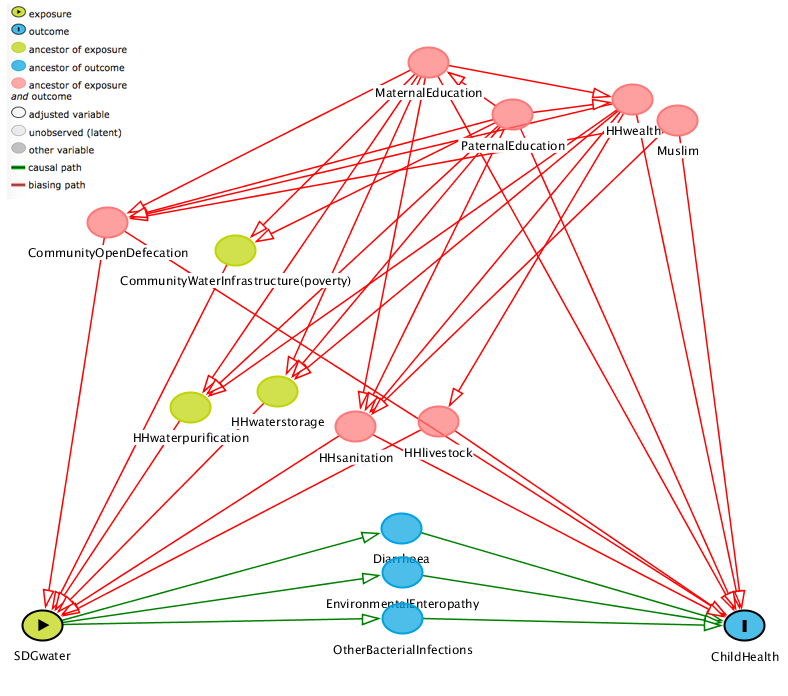


Arrows denote causal links; the directionality of arrows implies cause and effect.

**Supplemental Figure 1.** This Directed Acyclic Graph presents the minimum set of individual-level variables whose control is necessary to identify the causal relationship between household drinking water meeting SDG 6.1 standards and child health (Model: maternal education, paternal education, household socioeconomic status, religion of head of household, individual exposure to community open defecation, individual exposure to community poverty, household sanitation, household livestock).[1]

**References**

1. Textor J, Hardt J, Knuppel S. DAGitty: a graphical tool for analyzing causal diagrams. Epidemiology. 2011;22(5):745. doi: 10.1097/EDE.0b013e318225c2be. PubMed PMID: 21811114.
