# Supplementary material for: Effects of improved drinking water quality on early childhood growth in rural Uttar Pradesh, India: A propensity-score analysis
Source: PLoS One. 2019 Jan 8;14(1):e0209054. doi: 10.1371/journal.pone.0209054 (PMC6324831; doi:10.1371/journal.pone.0209054)
Supplement: S1 Methods — (DOCX) [file pone.0209054.s009.docx]

**Supplemental Methods: Variables and causal pathways included in the Directed Acyclic Graph (DAG)**

The DAG is a conceptual model describing how household drinking water is thought to affect childhood growth and survival. Nodes represent exposures or outcomes. Within the DAG framework, arrows denote causation (i.e. a change in the level or occurrence of the exposure leads to a change in the level or occurrence of the outcome).[1]

We used DAGs for three purposes: (1) To identify variables that require adjustment in the analysis; (2) To avoid adjusting for variables in cases where adjustment may introduce bias by creating conditional statistical associations between variables (i.e. by conditioning on the common outcome of two variables (known as ″colliders″), thereby introducing a form of selection bias); (3) To make explicit researchers′ understanding of causal relationships and to identify potential limitations in data and knowledge. Selection of variables and causal pathways was based on reviews and research studies on child nutrition and survival. Daggity statistical software was used to facilitate the procedure of choosing adjustment sets; that is, to identify a limited set of potential confounders as well as to avoid adjustment that may introduce bias through creating conditional associations (“backdoor paths”).[2] Importantly, our DAG identified household livestock as a potential confounding variable, for which data were not collected.

Our DAG is explicitly conceptualized within a multilevel framework to account for non-independence of outcomes at the village level. Following Fleischer and Diez-Roux (2007)[3], the DAGs we present are individual-level DAGs (the units of analysis are individuals and all variables are measured for each individual). We make simplifying assumptions of no heterogeneity of individual effects across higher-level units, and no cross-level interactions.[3]

**References**

1. Elwert F. Graphical Causal Models. In: Morgan SL, editor. Handbook of Causal Analysis for Social Research. Handbooks of Sociology and Social Research. Dordrecht, the Netherlands: Springer Science + Business Media; 2013. p. 29.

2. Textor J, Hardt J, Knuppel S. DAGitty: a graphical tool for analyzing causal diagrams. Epidemiology. 2011;22(5):745. doi: 10.1097/EDE.0b013e318225c2be. PubMed PMID: 21811114.

3. Fleischer NL, Diez Roux AV. Using directed acyclic graphs to guide analyses of neighbourhood health effects: an introduction. J Epidemiol Community Health. 2008;62(9):842-6. doi: 10.1136/jech.2007.067371.
