# Supplementary material for: Effects of improved drinking water quality on early childhood growth in rural Uttar Pradesh, India: A propensity-score analysis
Source: PLoS One. 2019 Jan 8;14(1):e0209054. doi: 10.1371/journal.pone.0209054 (PMC6324831; doi:10.1371/journal.pone.0209054)
Supplement: S2 File — Stata code used for analysis. (DOCX) [file pone.0209054.s011.docx]

********************************************

* PS_WATER ANALYSES *

********************************************/

version 15

clear

use pswater_rural.dta

capture log close

set more off

log using “filename", replace

/* Project: Propensity Score water analyses */

/* Author: Mira Johri*/

/* Date: updated 2018-08-25*/

/* Location: "filepath" */

numlabel, a

/* CREATE PROPENSITY SCORE USING LOGISTIC REGRESSION */

logistic sdg_water prop_opendef improved_sanitation i.Q_wealth_index i.educ_mother_4cat i.educ_father_4cat

muslim prop_poorest

predict ps

/* VERIFY PS OVERLAP (CONFOUNDERS-ONLY MODEL)*/

by sdg_water, sort: sum ps

/*

-> sdg_water = 0

Variable Obs Mean Std. Dev. Min Max

ps 652 .3885387 .0909422 .2410311 .7667052

-> sdg_water = 1

Variable Obs Mean Std. Dev. Min Max

ps 444 .4294432 .1055455 .2480777 .7466387*/

drop if ps <0.2480777

drop if ps >0.7466387

** Dropped 8 observations

/* VERIFY PS OVERLAP (FULL MODELS)*/

logistic sdg_water prop_opendef improved_sanitation i.Q_wealth_index i.educ_mother_4cat ///

i.educ_father_4cat muslim prop_poorest girl i.birthorder age

predict psn

by sdg_water, sort: sum psn

/*

---------------------------------------------------------------------------------------------------------------------

-> sdg_water = 0

Variable | Obs Mean Std. Dev. Min Max

-------------+---------------------------------------------------------

psn | 650 .3869978 .0948794 .2115978 .7655692

---------------------------------------------------------------------------------------------------------------------

-> sdg_water = 1

Variable | Obs Mean Std. Dev. Min Max

-------------+---------------------------------------------------------

psn | 443 .4321703 .1107669 .225306 .7923474

*/

drop if psn <0.225306

drop if psn >0.7655692

** Dropped 5 observations

//CREATE WEIGHTS BY HAND

gen ate_w=1/(1-ps) if sdg_water==0

replace ate_w=1/ps if sdg_water==1

gen att_w=ps/(1-ps) if sdg_water==0

replace att_w=1 if sdg_water==1

sum ate_w, det

sum att_w, det

/*CREATE DESCRIPTIVES TABLE*/

/*Table 1 Rural*/

tab1 sdg_water underweight stunting wasting

tab1 improved_sanitation Q_wealth_index muslim educ_mother_4cat educ_father_4cat girl birthorder

local outcomes " stunting underweight wasting"

foreach x of local outcomes {

tab `x' sdg_water, r chi

}

local candvar "improved_sanitation Q_wealth_index muslim motherage_cat educ_mother_4cat educ_father_4cat girl birthorder "

foreach x of local candvar {

tab `x' sdg_water, r chi

}

sum prop_opendef prop_poorest motherageR age

bysort sdg_water: sum prop_opendef prop_poorest motherageR age

/*CREATE AGE SPLINES*/

mkspline agespline = age, cubic nknots(5)

mkspline motheragespline = motherageR, cubic nknots(5)

/*MORE DESCRIPTIVES*/

tab1 open_defection purifywater

/*BALANCE DIAGNOSTICS FOR THE CONFOUNDER-ONLY MODEL*/

/* BALANCE DIAGNOSTICS*/

quietly teffects ipw (stunting) (sdg_water prop_opendef improved_sanitation i.Q_wealth_index i.educ_mother_4cat

i.educ_father_4cat muslim prop_poorest, logit), vce(robust)

tebalance summarize

/* OVERLAP GRAPH*/

teffects overlap, ptlevel(1)

/*IMAI OVERIDENTIFICATION TEST FOR COVARIATE BALANCE

tebalance overid

This is the confounders only model. The Imai test could not be computed in the presence of the age spline variable, but results with the other variables are consistent with the null hypothesis that covariates are balanced. ///

Results are the same for all outcomes. */

quietly teffects ipw (stunting) (sdg_water prop_opendef improved_sanitation i.Q_wealth_index i.educ_mother_4cat

i.educ_father_4cat muslim prop_poorest, logit), vce(robust)

tebalance overid

/*Overidentification test for covariate balance

H0: Covariates are balanced:

chi2(15) = 12.2488

Prob > chi2 = 0.6601

*/

************************************************************************x

/* CONFOUNDER-ONLY MODEL AND ALL OUTCOMES */

************************************************************************/

local y "stunting underweight wasting"

set more off

foreach var in `y' {

teffects ipw (`var') (sdg_water prop_opendef improved_sanitation i.Q_wealth_index

i.educ_mother_4cat i.educ_father_4cat muslim prop_poorest, logit), vce(robust)

teffects ipw (`var') (sdg_water prop_opendef improved_sanitation i.Q_wealth_index

i.educ_mother_4cat i.educ_father_4cat muslim prop_poorest, logit), vce(robust)

aequations pomeans

teffects ipw (`var') (sdg_water prop_opendef improved_sanitation i.Q_wealth_index

i.educ_mother_4cat i.educ_father_4cat muslim prop_poorest, logit), vce(robust) coefleg

nlcom _b[ATE:r1vs0.sdg_water]/_b[POmean:0.sdg_water]

}

************************************************************************x

/* RECALCULATE USING SURVEY WEIGHTS (For comparison) */

************************************************************************/

local y "stunting underweight wasting"

set more off

foreach var in `y' {

logit `var' i.sdg_water [pw=ate_w], vce(robust) or

margins sdg_water, post

lincom (_b[1.sdg_water]-_b[0.sdg_water])

}

************************************************************************x

/* BALANCE DIAGNOSTICS FULL MODELS: IPW */

************************************************************************/

/* BALANCE DIAGNOSTICS*/

quietly teffects ipw (stunting) (sdg_water prop_opendef improved_sanitation i.Q_wealth_index

i.educ_mother_4cat i.educ_father_4cat muslim prop_poorest girl ///

i.birthorder i.girl#i.birthorder agespline*, logit), vce(robust)

/* OVERLAP GRAPH*/

teffects overlap, ptlevel(1)

/*STD MEAN DIFFERENCES*/

tebalance summarize

************************************************************************x

/* ATE: EFFECTS ON ALL NUTRITIONAL OUTCOMES */

************************************************************************/

local y "stunting underweight wasting"

set more off

foreach var in `y' {

teffects ipw (`var') (sdg_water prop_opendef improved_sanitation i.Q_wealth_index

i.educ_mother_4cat i.educ_father_4cat muslim prop_poorest ///

girl i.birthorder i.girl#i.birthorder agespline*, logit), vce(robust)

teffects ipw (`var') (sdg_water prop_opendef improved_sanitation i.Q_wealth_index

i.educ_mother_4cat i.educ_father_4cat muslim prop_poorest ///

girl i.birthorder i.girl#i.birthorder agespline*, logit), vce(robust) aequations pomeans

teffects ipw (`var') (sdg_water prop_opendef improved_sanitation i.Q_wealth_index

i.educ_mother_4cat i.educ_father_4cat muslim prop_poorest ///

girl i.birthorder i.girl#i.birthorder agespline*, logit), vce(robust) coefleg

nlcom _b[ATE:r1vs0.sdg_water]/_b[POmean:0.sdg_water]

}

************************************************************************x

/* ATE: EFFECTS ON ALL NUTRITIONAL OUTCOMES - CONTINUOUS */

************************************************************************/

local z "HAZ WAZ WHZ"

set more off

foreach var in `z' {

teffects ipw (`var') (sdg_water prop_opendef improved_sanitation i.Q_wealth_index

i.educ_mother_4cat i.educ_father_4cat muslim prop_poorest, logit), vce(robust)

}

local y "HAZ WAZ WHZ"

set more off

foreach var in `y' {

teffects ipw (`var') (sdg_water prop_opendef improved_sanitation i.Q_wealth_index

i.educ_mother_4cat i.educ_father_4cat muslim prop_poorest ///

girl i.birthorder i.girl#i.birthorder agespline*, logit), vce(robust)

}

************************************************************************x

/* ATT: CONFOUNDER-ONLY MODELS */

************************************************************************/

/*CONFOUNDER-ONLY MODEL AND ALL OUTCOMES*/

local y "stunting underweight wasting"

set more off

foreach var in `y' {

teffects ipw (`var') (sdg_water prop_opendef improved_sanitation i.Q_wealth_index

i.educ_mother_4cat i.educ_father_4cat muslim prop_poorest, logit), atet vce(robust)

teffects ipw (`var') (sdg_water prop_opendef improved_sanitation i.Q_wealth_index

i.educ_mother_4cat i.educ_father_4cat muslim prop_poorest, logit), atet vce(robust)

coefleg

*nlcom _b[ATET:r1vs0.sdg_water]/_b[POmean:0.sdg_water]

}

************************************************************************x

/* ATT: FULL MODELS */

************************************************************************/

local y "stunting underweight wasting"

set more off

foreach var in `y' {

teffects ipw (`var') (sdg_water prop_opendef improved_sanitation i.Q_wealth_index

i.educ_mother_4cat i.educ_father_4cat muslim prop_poorest ///

girl i.birthorder i.girl#i.birthorder agespline*, logit), atet vce(robust)

teffects ipw (`var') (sdg_water prop_opendef improved_sanitation i.Q_wealth_index

i.educ_mother_4cat i.educ_father_4cat muslim prop_poorest ///

girl i.birthorder i.girl#i.birthorder agespline*, logit), atet vce(robust) aequations

teffects ipw (`var') (sdg_water prop_opendef improved_sanitation i.Q_wealth_index

i.educ_mother_4cat i.educ_father_4cat muslim prop_poorest ///

girl i.birthorder i.girl#i.birthorder agespline*, logit), atet vce(robust) coefleg

*nlcom _b[ATET:r1vs0.sdg_water]/_b[POmean:0.sdg_water]

}

************************************************************************x

/* GRAPH COMBINE: FIGURE 1 */

************************************************************************/

*graph combine psdensity_confounders.gph psdensity_full.gph, col(1) iscale(1)

************************************************************************x

/* SENSITIVITY ANALYSIS: UNADJUSTED EFFECTS */

************************************************************************/

local outcomes " stunting underweight wasting"

foreach x of local outcomes {

logit `x' sdg_water

}

/* Assess crude associations using GEE regression*/

xtset id_cluster

xtgee sdg_water, family(binomial) link(logit) vce (robust) corr(exchangeable)

xtgee underweight sdg_water, family(binomial) link(logit) vce (robust) corr(exchangeable)

xtgee stunting sdg_water, family(binomial) link(logit) vce (robust) corr(exchangeable)

xtgee wasting sdg_water, family(binomial) link(logit) vce (robust) corr(exchangeable)

************************************************************************

/* SENSITIVITY ANALYSIS: REGRESSION-ADJUSTED EFFECTS */

************************************************************************/

//with confounders only for each outcome

xtset id_cluster

xtgee stunting sdg_water prop_opendef improved_sanitation i.Q_wealth_index

i.educ_mother_4cat i.educ_father_4cat muslim prop_poorest, family(binomial) link(logit) vce (robust) corr(exchangeable)

xtgee underweight sdg_water prop_opendef improved_sanitation i.Q_wealth_index

i.educ_mother_4cat i.educ_father_4cat muslim prop_poorest, family(binomial) link(logit) vce (robust) corr(exchangeable)

xtgee wasting sdg_water prop_opendef improved_sanitation i.Q_wealth_index

i.educ_mother_4cat i.educ_father_4cat muslim prop_poorest, family(binomial) link(logit)

vce (robust) corr(exchangeable)

//with full models for each outcome

xtgee stunting sdg_water prop_opendef improved_sanitation i.Q_wealth_index

i.educ_mother_4cat i.educ_father_4cat muslim prop_poorest i.girl i.birthorder

i.girl#i.birthorder agespline*, family(binomial) link(logit) vce (robust) corr(exchangeable)

xtgee underweight sdg_water prop_opendef improved_sanitation i.Q_wealth_index

i.educ_mother_4cat i.educ_father_4cat muslim prop_poorest i.girl i.birthorder

i.girl#i.birthorder agespline*, family(binomial) link(logit) vce (robust) corr(exchangeable)

xtgee wasting sdg_water prop_opendef improved_sanitation i.Q_wealth_index

i.educ_mother_4cat i.educ_father_4cat muslim prop_poorest i.girl i.birthorder i.girl#i.birthorder agespline*, family(binomial) link(logit) vce (robust) corr(exchangeable)

************************************************************************x

/* SENSITIVITY ANALYSIS TRUNCATING SURVEY WEIGHTS */

************************************************************************/

//CREATE WEIGHTS BY HAND

gen aten_w=1/(1-psn) if sdg_water==0

replace aten_w=1/psn if sdg_water==1

sum aten_w

sum aten_w, det

***Nutritional Outcomes*****

sort aten_w

su aten_w, det

count if aten_w <1.334924

count if aten_w >3.858561

gen aten_wt1=aten_w

replace aten_wt1=1.334924 in 1/11

di 1088-11

replace aten_wt1=3.858561 in 1078/1088

count if aten_w <1.395667

count if aten_w > 3.264823

gen aten_wt5 = aten_w

replace aten_wt5 = 1.395667 in 1/54

di 1088-54

replace aten_wt5 = 3.264823 in 1034/1088

su aten_w aten_wt1 aten_wt5

list aten_w aten_wt1 aten_wt5 in 1/56

list aten_w aten_wt1 aten_wt5 in 1033/1088

local y "stunting underweight wasting"

set more off

foreach var in `y' {

logit `var' i.sdg_water [pw=aten_wt1], vce(robust) or

margins sdg_water, post

lincom (_b[1.sdg_water]-_b[0.sdg_water])

}

local z "stunting underweight wasting"

set more off

foreach var in `z' {

logit `var' i.sdg_water [pw=aten_wt5], vce(robust) or

margins sdg_water, post

lincom (_b[1.sdg_water]-_b[0.sdg_water])

}

************************************************************************x

/* RECALCULATE USING IPWRA Models (For comparison) */

************************************************************************/

teffects ipwra (stunting prop_opendef improved_sanitation i.Q_wealth_index

i.educ_mother_4cat i.educ_father_4cat muslim prop_poorest i.girl i.birthorder

i.girl#i.birthorder agespline* , logit) ///

(sdg_water prop_opendef improved_sanitation i.Q_wealth_index i.educ_mother_4cat i.educ_father_4cat muslim prop_poorest), vce(robust)

teffects ipwra (underweight prop_opendef improved_sanitation i.Q_wealth_index i.educ_mother_4cat

i.educ_father_4cat muslim prop_poorest i.girl i.birthorder i.girl#i.birthorder agespline* , logit) ///

(sdg_water prop_opendef improved_sanitation i.Q_wealth_index i.educ_mother_4cat i.educ_father_4cat muslim prop_poorest), vce(robust)

teffects ipwra (wasting prop_opendef improved_sanitation i.Q_wealth_index i.educ_mother_4cat i.educ_father_4cat

muslim prop_poorest i.girl i.birthorder i.girl#i.birthorder agespline* , logit) ///

(sdg_water prop_opendef improved_sanitation i.Q_wealth_index i.educ_mother_4cat i.educ_father_4cat muslim prop_poorest), vce(robust)

************************************************************************x

/* RECALCULATE USING PSmatch Models (For comparison) */

************************************************************************/

/*Confounder-only models*/

teffects psmatch (stunting) (sdg_water prop_opendef improved_sanitation i.Q_wealth_index i.educ_mother_4cat

i.educ_father_4cat muslim prop_poorest,logit), vce(robust) caliper(0.15)

tebalance box

tebalance density

teffects psmatch (underweight) (sdg_water prop_opendef improved_sanitation i.Q_wealth_index i.educ_mother_4cat i.educ_father_4cat muslim prop_poorest,logit), vce(robust) caliper(0.15)

tebalance box

tebalance density

teffects psmatch (wasting) (sdg_water prop_opendef improved_sanitation i.Q_wealth_index i.educ_mother_4cat

i.educ_father_4cat muslim prop_poorest,logit), vce(robust) caliper(0.15)

tebalance box

tebalance density

/*Fully adjusted models */

teffects psmatch (stunting) (sdg_water prop_opendef improved_sanitation i.Q_wealth_index i.educ_mother_4cat

i.educ_father_4cat muslim prop_poorest ///

i.girl i.birthorder i.girl#i.birthorder age,logit), vce(robust) caliper(0.15)

tebalance box

tebalance density

teffects psmatch (underweight) (sdg_water prop_opendef improved_sanitation i.Q_wealth_index i.educ_mother_4cat

i.educ_father_4cat muslim prop_poorest ///

i.girl i.birthorder i.girl#i.birthorder age,logit), vce(robust) caliper(0.15)

tebalance box

tebalance density

teffects psmatch (wasting) (sdg_water prop_opendef improved_sanitation i.Q_wealth_index i.educ_mother_4cat

i.educ_father_4cat muslim prop_poorest ///

i.girl i.birthorder i.girl#i.birthorder age,logit), vce(robust) caliper(0.15)

tebalance box

tebalance density

/*

/*Fully adjusted models no caliper for comparison */

teffects psmatch (stunting) (sdg_water prop_opendef improved_sanitation i.Q_wealth_index i.educ_mother_4cat

i.educ_father_4cat muslim prop_poorest ///

i.girl i.birthorder i.girl#i.birthorder agespline* ,logit), vce(robust)

teffects psmatch (underweight) (sdg_water prop_opendef improved_sanitation i.Q_wealth_index i.educ_mother_4cat i.educ_father_4cat muslim prop_poorest ///

i.girl i.birthorder i.girl#i.birthorder agespline* ,logit), vce(robust)

teffects psmatch (wasting) (sdg_water prop_opendef improved_sanitation i.Q_wealth_index i.educ_mother_4cat

i.educ_father_4cat muslim prop_poorest ///

i.girl i.birthorder i.girl#i.birthorder agespline* ,logit), vce(robust)

/* without interaction */

teffects psmatch (stunting) (sdg_water prop_opendef improved_sanitation i.Q_wealth_index i.educ_mother_4cat

i.educ_father_4cat muslim prop_poorest ///

i.girl i.birthorder agespline* ,logit), vce(robust) caliper(0.15)

teffects psmatch (underweight) (sdg_water prop_opendef improved_sanitation i.Q_wealth_index i.educ_mother_4cat

i.educ_father_4cat muslim prop_poorest ///

i.girl i.birthorder agespline* ,logit), vce(robust) caliper(0.15)

teffects psmatch (wasting) (sdg_water prop_opendef improved_sanitation i.Q_wealth_index i.educ_mother_4cat

i.educ_father_4cat muslim prop_poorest ///

i.girl i.birthorder agespline* ,logit), vce(robust) caliper(0.15)

*/

clear

exit
