# Supplementary material for: Effects of improved drinking water quality on early childhood growth in rural Uttar Pradesh, India: A propensity-score analysis
Source: PLoS One. 2019 Jan 8;14(1):e0209054. doi: 10.1371/journal.pone.0209054 (PMC6324831; doi:10.1371/journal.pone.0209054)
Supplement: S3 File — Questionnaire used for data collection in Hindi. (PDF) [file pone.0209054.s012.pdf]

फॉर्म संख्या-१

हॉउसहोल्ड रोस्टर के लिए

	**पहचान**		
1	परिवार संख्या<br>**(घर में भरने के लिए)**		
2	साक्षात्कार की तारीख	**दिन** / **माह** / **वर्ष**	
3	साक्षातकर्ता का नाम		
4	राज्य	यूपी ---<br>दिल्ली ---------------------------------------	1<br>2
5	जिला	हरदोई--<br>कीर्ति नगर ----------------------------------	1<br>2
6	ब्लॉक		
7	गांव/क्लस्टर का नाम और कोड		**गाँव/क्लस्टर कोड**
8	घर का फ़ोन नंबर (घर के किसी भी सदस्य का फ़ोन नंबर लिखा जा सकता है)		

घरेलू अनुसूची

**प्र. सं.**	**प्रश्न**	**उत्तर**	**कोड***	**में जाएँ**
***नोट– कोड कॉलम में अंकीय कोड एक उत्तर और वर्णमाला कोड विविध उत्तरों के लिए है**				
1	घर मे रहने वाले सदस्यों की कुल संख्या (जो घर मे एक चूल्हे से बना खाना खाते हो)			
2	घर का प्रकार<br>**(केवल एक उत्तर मान्य)**	एकल--<br>संयुक्त--	१<br>२	
3	घर के मुखिया का धर्म<br>**(केवल एक उत्तर मान्य)**	हिंदू--<br>मुसलमान--<br>सिक्ख---<br>ईसाइ---<br>अन्य--	१<br>२<br>३<br>४<br>५	
**(निर्देश: यदि घर मिटी के गारे या अन्य निम्न कोटि की सामग्री से बना है तो यह कच्चा घर है. यदि घर आंशिक रूप से निम्नकोटि का बना है और आंशिक रूप से उच्चकोटि का तो इसे आधा पक्का में वर्गीकृत करें और यदि घर में छत, दीवार और फर्श में पूरी तरह उच्चकोटि की सामग्री लगी है तो इसे पक्का के बतौर वर्गीकृत करेंगे.)**				
4	घर का प्रकार	कच्चा--	१	

	**(नोट- कृपया देखें और कोड डालें)**<br>**(केवल एक उत्तर मान्य)**	आधा पक्का--<br>पक्का--	२<br>३	
5	घर में कुल कमरों की संख्या			
6	घर का फर्श<br>**(नोट- कृपया देखें और कोड डालें)**<br>**(विविध उत्तर मान्य)**	गन्दा/रेत/गोबर वाला------------------------------------<br>बेहतर फर्श (सीमेंट/पत्थर/ईंट)-------------------------<br>अन्य ---	a<br>b<br>c	
7	शौचालय व्यवस्था<br>**(विविध उत्तर मान्य)**	खुले में ---<br>सामुदायिक शौचालय व्यवस्था-----------------------<br>घर में शौचालय व्यवस्था------------------------------	a<br>b<br>c	
8	खाना बनाने का ईंधन<br>**(नोट- कृपया देखें और कोड डालें)**<br>**(विविध उत्तर मान्य)**	गोबर के उपले--<br>लकड़ी--<br>चारकोल---<br>एलपीजी सिलेंडर--<br>मिट्टी का तेल ---<br>बिजली का हीटर---------------------------------------<br>अन्य ---	a<br>b<br>c<br>d<br>e<br>f<br>g	
9	पेयजल का स्रोत<br>**(विविध उत्तर मान्य)**	टेप का पानी---<br>हैण्डपम्प--<br>कुआँ---<br>सतह का पानी--<br>पानी का टेंकर--<br>अन्य--	a<br>b<br>c<br>d<br>e<br>f	
10	क्या आप पानी को पीने से पहले उसे शुद्ध करने के लिए कुछ करते हैं?<br>**(केवल एक उत्तर मान्य)**	हां---<br>नहीं---<br>नहीं मालूम---	१<br>२<br>३	12
11	यदि हां तो क्या करते हैं?<br>**(विविध उत्तर मान्य)**	उबालना---<br>क्लोरीन की गोली डालना------------------------------<br>कपडे से छानते हैं -------------------------------------<br>वाटर फ़िल्टर का प्रयोग करते हैं------------------<br>कुछ नहीं करते ---<br>अन्य ---	a<br>b<br>c<br>d<br>e<br>f	
12	क्या आपकी खेती की जमीन है?	हां---	१	

	**(केवल एक उत्तर मान्य)**	नहीं--	२	
13	घरेलू संपत्ति<br><br>**(विविध उत्तर मान्य)**	रेडियो--<br>साईकिल---<br>कार---<br>मोटरसाइकिल/स्कूटर-----------------------------------<br>मोबाइल फोन ---<br>टेलीफोन (लेंडलाइन)-----------------------------------<br>बिजली---<br>टीवी---<br>रेफ्रिजरेटर --	a<br>b<br>c<br>d<br>e<br>f<br>g<br>h<br>i	
14	घर में ऐसी कितनी महिलायें हैं (एक ही चूल्हे में खाने वाली) जिनका 12 - 23 महीने का बच्चा है?			

(रिमाइंडर: फॉर्म २ प्रत्येक उस महिला के लिए भरा जाएगा जिसका कि 12 - 23 माह का बच्चा हो - प्रश्न 14 में लिखी महिलाओं की संख्या के अनुसार)

शीट - 1 (एक घर मे प्रथम योग्य महिला के लिए)

प्रश्नों के नीचे दी गई जगह में उत्तर कोड भरें															
**नोट - नीचे के प्रश्न केवल उन परिवारों के लिए लागू हैं जिनके पास १२ से २३ माह का बच्चा है**															
ला इन न.	नाम	रिश्ता	निवास	लिंग	उम्र		शिक्षा (यदि उम्र 5 सालसे कम है तो NA लिखें)			रोजगार को स्थिति					
15	16	17*	18	19	20	21	22	23.a	23.b	24					
	सभी व्यक्तियों का नाम लिखें जो कि आपके घर में रहते हैं (योग्य महिला, उसका पति और उनके बच्चे) योग्य महिला से शुरू करे	योग्य महिला से क्या रिश्ता है?	क्या सामान्यत या यहीं रहता/रहती है?<br>हां-1<br>नहीं-2	क्या (नाम) पुरुष है या स्त्री<br>पुरुष - 1<br>स्त्री - 2	केवल 2 साल से अधिक उम्र वाले सदस्यों की उम्र पूण वर्षों मे लिखें.	केवल 2 साल से छोटे शिशु की उम्र महीनों में लिखें	क्या कभी स्कूल गया है?<br>हां – 1<br>नहीं – 2<br>**यदि नहीं तो सेल सं.** 24 **में जाएँ**	यदि अभी स्कूल में है<br>वर्तमान स्थिति लिखें	यदि अभी स्कूल में नहीं है<br>(नाम) की सबसे ऊचीं शैक्षिक योग्यता?	दैनिक वेतन	अपनी ज़मीन पर काम	निश्चित आय	स्वरोज गार	गृहिणी	बेरोजगार
1															
2															
3															
4															
5															
6															
7															
8															
9															
10															

**योग्य महिला के साथ रिश्ता (17*के लिए)**	**योग्य महिला**	**पत्नी या पति**	**पुत्र**	**पुत्री**
**कोड**	**1**	**2**	**3**	**4**

शीट - 2 (एक घर मे दूसरी योग्य महिला के लिए)

प्रश्नों के नीचे दी गई जगह में उत्तर कोड भरें

नोट - नीचे के प्रश्न केवल उन परिवारों के लिए लागू हैं जिनके पास १२ से २३ माह का बच्चा है

ला इन न.	नाम	रिश्ता	निवास	लिंग	उम्र		शिक्षा (यदि उम्र 5 सालसे कम है तो NA लिखें)			रोजगार को स्थिति					
15	16	17*	18	19	20	21	22	23.a	23.b	24					
	सभी व्यक्तियों का नाम लिखें जो कि आपके घर में रहते हैं (योग्य महिला, उसका पति और उनके बच्चे) योग्य महिला से शुरू करे	योग्य महिला से क्या रिश्ता है?	क्या सामान्यत या यहीं रहता/रहती है?<br>हां-1<br>नहीं-2	क्या (नाम) पुरुष है या स्त्री<br>पुरुष - 1<br>स्त्री - 2	केवल 2 साल से अधिक उम्र वाले सदस्यों की उम्र पूण वर्षों मे लिखें.	केवल 2 साल से छोटे शिशु की उम्र महीनों में लिखें	क्या कभी स्कूल गया है?<br>हां – 1<br>नहीं – 2<br>**यदि नहीं तो सेल सं.** 24 **में जाएँ**	यदि अभी स्कूल में है<br>वर्तमान स्थिति लिखें	यदि अभी स्कूल में नहीं है<br>(नाम) की सबसे ऊचीं शैक्षिक योग्यता?	दैनिक वेतन	अपनी ज़मीन पर काम	निश्चित आय	स्वरोज गार	गृहिणी	बेरोजगार
11															
12															
13															
14															
15															
16															
17															
18															
19															
20															

**योग्य महिला के साथ रिश्ता (17*के लिए)**	**योग्य महिला**	**पत्नी या पति**	**पुत्र**	**पुत्री**
**कोड**	**1**	**2**	**3**	**4**

शीट - 3 (एक घर मे तीसरी योग्य महिला के लिए)

प्रश्नों के नीचे दी गई जगह में उत्तर कोड भरें

नोट - नीचे के प्रश्न केवल उन परिवारों के लिए लागू हैं जिनके पास १२ से २३ माह का बच्चा है

लाइन न.	नाम	रिश्ता	निवास	लिंग	उम्र		शिक्षा (यदि उम्र 5 सालसे कम है तो NA लिखें)			रोजगार को स्थिति					
15	16	17*	18	19	20	21	22	23.a	23.b	24					
	सभी व्यक्तियों का नाम लिखें जो कि आपके घर में रहते हैं (योग्य महिला, उसका पति और उनके बच्चे) योग्य महिला से शुरू करे	योग्य महिला से क्या रिश्ता है?	क्या सामान्यत या यहीं रहता/रहती है?<br>हां-1<br>नहीं-2	क्या (नाम) पुरुष है या स्त्री<br>पुरुष - 1<br>स्त्री - 2	केवल 2 साल से अधिक उम्र वाले सदस्यों की उम्र पूण वर्षों मे लिखें.	केवल 2 साल से छोटे शिशु की उम्र महीनों में लिखें	क्या कभी स्कूल गया है?<br>हां – 1<br>नहीं – 2<br>**यदि नहीं तो सेल सं.** 24 **में जाएँ**	यदि अभी स्कूल में है<br>वर्तमान स्थिति लिखें	यदि अभी स्कूल में नहीं है<br>(नाम) की सबसे ऊचीं शैक्षिक योग्यता?	दैनिक वेतन	अपनी ज़मीन पर काम	निश्चित आय	स्वरोज गार	गृहिणी	बेरोजगार
21															
22															
23															
24															
25															
26															
27															
28															
29															
30															

**योग्य महिला के साथ रिश्ता (17*के लिए)**	**योग्य महिला**	**पत्नी या पति**	**पुत्र**	**पुत्री**
**कोड**	**1**	**2**	**3**	**4**

निर्देश - उचित उत्तर कोड को गोल करें

25.	**प्रश्न**	**उत्तर**	**कोड**
	फीकल कोलीफोर्म बैक्टीरिया के लिए पानी के परीक्षण के परिणाम (कृपया गोला करें, केवल एक उत्तर मान्य है)	दूषित ----------------------------------<br>दूषित नहीं-----------------------------	१<br>२
