# Supplementary material for: Effects of improved drinking water quality on early childhood growth in rural Uttar Pradesh, India: A propensity-score analysis
Source: PLoS One. 2019 Jan 8;14(1):e0209054. doi: 10.1371/journal.pone.0209054 (PMC6324831; doi:10.1371/journal.pone.0209054)
Supplement: S4 File — English translation of questionnaire used for data collection. (PDF) [file pone.0209054.s013.pdf]

Form No - 1

For the Household Roster

Identification		
1	Household Number<br>**(To be filled at household)**	
2	Date of Interview	Day / Month / Year
3	Name of the Interviewer	
4	State	Uttar Pradesh------------------------------- 1<br>Delhi--- 2
5	District	Hardoi-- 1<br>Kirti Nagar---------------------------------- 2
6	Block	
7	Village/ cluster name & Code	**Village/Cluster Code**
8	Phone Number of the household (Any household member phone number)	

Household Schedule

Q.No	Question	Responses	Code*	Skip to
	***Note – In the code column numeric codes are for the Single response and Alphabetic codes are for multiple responses**			
1	Total number of people in the family living in this household (eating from the same chulha)			
2	Type of Family<br>**(Only single response allowed)**	Nuclear---<br>Joint--	1<br>2	
3	Religion of the head of household<br>**(Only single response allowed)**	Hindu---<br>Muslim---<br>Sikh---<br>Christian---<br>Other---	1<br>2<br>3<br>4<br>5	
	**(Instruction: If the house is made of mud thatch or other low quality materials it is a kachcha house. If the house is made of partly low quality and partly high quality materials classify it as semi-pucca and if houses made of high quality materials throughout, including the roof, walls and floor classify it as pucca.)**			
4	Type of Household<br>**(Note- Please observe and code)**<br>**(Only single response allowed)**	Kachcha---<br>Semi pucca--<br>Pucca---	1<br>2<br>3	
5	Total number of rooms in the house			
6	Flooring in the household<br>**(Note- Please observe and code)**<br>**(Multiple responses allowed)**	Dirt/Sand/Dung---------------------------------------<br>Improved flooring (Cement/Stone/Brick)----------<br>Other ---	a<br>b<br>c	

7	Toilet Facility<br><br>**(Multiple responses allowed)**	Open defecation--<br>Community toilet facility------------------------------<br>Toilet facility at home----------------------------------	a<br>b<br>c	
8	Cooking Fuel<br>**(Note-Please observe and code)**<br><br>**(Multiple responses allowed)**	Dung---<br>Wood---<br>Charcoal--<br>LPG cylinder---<br>Kerosene oil---<br>Electric heater---<br>Other --	a<br>b<br>c<br>d<br>e<br>f<br>g	
9	Source of drinking water<br><br>**(Multiple responses allowed)**	Tap water--<br>Hand pump---<br>Well--<br>Surface water---<br>Water tanker--<br>Other--	a<br>b<br>c<br>d<br>e<br>f	
10	Do you do anything to purify water before drinking?<br><br>**(Only single response allowed)**	Yes--<br>No---<br>Don't Know---	1<br>2<br>3	2, 3 → 12
11	If yes, what do you do with the water before drinking?<br><br>**(Multiple responses allowed)**	Boiling--<br>Add chlorine tab/alum---------------------------------<br>Filter through cloth-------------------------------------<br>Use water filter--<br>Don't do anything --------------------------------------<br>Other --	a<br>b<br>c<br>d<br>e<br>f	
12	Do you own land for cultivation?<br><br>**(Only single response allowed)**	Yes--<br>No---	1<br>2	
13	Household possessions<br><br>**(Multiple responses allowed)**	Radio--<br>Bicycle--<br>Car---<br>Motorcycle/Scooter------------------------------------<br>Mobile phone ---<br>Telephone (landline)-----------------------------------<br>Electricity ---<br>Television---<br>Refrigerator---	a<br>b<br>c<br>d<br>e<br>f<br>g<br>h<br>i	
14	How many women are there in the household **(eating from the same chulha)** with a child in the age group 12-23 months?			

(Reminder: Form 2 should be completed for <u>each</u> woman in the household with a child in the age group 12-23 months – that is, for the number of women given in question 14).

Sheet – 1 (For the First Eligible Mother in a household)

Please fill response code in given space below the questions

Note – Questions below are only pertinent to a family group having 12 to 23 months old child

Line No	Name	Relation	Residence	Sex	Age		Education (If age less than 5 year write NA)			Employment status					
15	16	17*	18	19	20	21	22	23.a	23.b	24					
	Write the name of all the persons usually living in your nuclear family (Eligible mother, her Husband, and their children only)	What is the relationship of (Name) to the Eligible mother	Does (Name) usually live here?<br><br>Yes-1<br>No-2	Is (Name) male or Female<br><br>Male – 1<br>Female - 2	How old is (Name)? **(Write in completed years for everyone. For infant<2 years, do not record the age here. Use cell 21)**	For infant<2 years only, Insert age in months	Has (Name) ever been to school?<br><br>Yes – 1<br>No – 2<br>**If No go to cell no 24**	If currently in school<br><br>Write current std.	If currently not in school<br><br>What is the highest completed educational qualification of (Name)?	Daily wages	Work on own land	Fixed Income	Self employed	House wife	Unemployed
1															
2															
3															
4															
5															
6															
7															
8															
9															
10															

**Relation with the Eligible Mother(For 17*)**	**Eligible Mother**	**Husband**	**Son**	**daughter**
**Code**	**1**	**2**	**3**	**4**

Sheet – 2 (For the Second Eligible Mother in a household)

Please fill response code in given space below the questions

Note – Questions below are only pertinent to a family group having 12 to 23 months old child

Line No	Name	Relation	Residence	Sex	Age		Education (If age less than 5 year write NA)			Employment status					
15	16	17*	18	19	20	21	22	23.a	23.b	24					
	Write the name of all the persons usually living in your nuclear family (Eligible mother, her Husband, and their children only)	What is the relationship of (Name) to the Eligible mother	Does (Name) usually live here?<br>Yes-1<br>No-2	Is (Name) male or Female<br>Male – 1<br>Female - 2	How old is (Name)? **(Write in completed years for everyone. For infant<2 years, do not record the age here. Use cell 21)**	For infant<2 years only, Insert age in months	Has (Name) ever been to school?<br>Yes – 1<br>No – 2<br>**If No go to cell no 24**	If currently in school<br>Write current std.	If currently not in school<br>What is the highest completed educational qualification of (Name)?	Daily wages	Work on own land	Fixed Income	Self employed	House wife	Unemployed
11															
12															
13															
14															
15															
16															
17															
18															
19															
20															

**Relation with the Eligible Mother(For 17*)**	**Eligible Mother**	**Husband**	**Son**	**daughter**
**Code**	**1**	**2**	**3**	**4**

Sheet – 3 (For the Third Eligible Mother in a household)

Please fill response code in given space below the questions

Note – Questions below are only pertinent to a family group having 12 to 23 months old child

Line No	Name	Relation	Residence	Sex	Age		Education (If age less than 5 year write NA)			Employment status					
15	16	17*	18	19	20	21	22	23.a	23.b	24					
	Write the name of all the persons usually living in your nuclear family (Eligible mother, her Husband, and their children only)	What is the relationship of (Name) to the Eligible mother	Does (Name) usually live here?<br>Yes-1<br>No-2	Is (Name) male or Female<br>Male – 1<br>Female - 2	How old is (Name)? **(Write in completed years for everyone. For infant<2 years, do not record the age here. Use cell 21)**	For infant<2 years only, Insert age in months	Has (Name) ever been to school?<br>Yes – 1<br>No – 2<br>**If No go to cell no 24**	If currently in school<br>Write current std.	If currently not in school<br>What is the highest completed educational qualification of (Name)?	Daily wages	Work on own land	Fixed Income	Self employed	House wife	Unemployed
21															
22															
23															
24															
25															
26															
27															
28															
29															
30															

**Relation with the Eligible Mother(For 17*)**	**Eligible Mother**	**Husband**	**Son**	**daughter**
**Code**	**1**	**2**	**3**	**4**

Instruction - Please circle appropriate response code

25.	Question	Response	Code
	Results of water testing for faecal coliform bacteria (please circle, only one response allowed)	Contaminated ---------------------------------<br>Not Contaminated-----------------------------	1<br>2
